# CDK2 inhibition promotes neuronal differentiation in neuroblastoma

**DOI:** 10.1038/s41598-026-38123-4

**Published:** 2026-02-06

**Authors:** Mohammad Alzrigat, Loay Mahmoud, Ada Nursel Topçu, Marta Valenti, Lu Zhang, Puck Veen, Fabian John, Wesam Bazzar, Kaisa Lehti, Lars-Gunnar Larsson

**Affiliations:** 1 Department of Microbiology, Tumor and Cell Biology, Karolinska Institutet, Solna, Stockholm, Sweden; 2 Department of Chemistry and Biomedical Science, Faculty of Natural Sciences, Norwegian University of Science and Technology, Trondheim, Norway; 3 Department of Pharmaceutical Biosciences, Uppsala Biomedical Centre, Uppsala University, Uppsala, Sweden; 4 Present Address: Institute for Neuroscience and Cardiovascular Research, The University of Edinburgh, Edinburgh, UK; 5 Present Address: Centre for Regenerative Medicine “Stefano Ferrari”, Department of Life Science, University of Modena and Reggio Emilia, Modena, Italy

**Keywords:** Neuroblastoma, CDK2, Neuronal differentiation, Cancer, Oncology

## Abstract

Neuroblastoma (NB) is a childhood tumor arising from neural crest-derived progenitor cells. Cyclin dependent kinase 2 (CDK2) has been suggested to be a promising therapeutic target in NB especially *MYCN*-amplified tumors. How CDK2 contributes to the development of NB is not fully understood. Here, we demonstrate that high CDK2 expression in NB correlates with advanced and high-risk disease, *MYCN*-amplification, poor prognosis, and undifferentiated tumors. We uncovered that CDK2 genetic or pharmacological inhibition induces neuronal differentiation in human NB cell lines, which effect is in general more prominent in the *MYCN*-amplified subtype. Further, we establish CDK2 as a MYCN target gene that modulate the MYC-pathway in *MYCN*-amplified NB. Notably, pharmacological inhibition of CDK2 in combination with MYCN inhibition or all-*trans*-retinoic acid (ATRA) differentiation therapy enhances neuronal differentiation in vitro. These results reveal an important function of CDK2 in NB and highlight CDK2 inhibition alone or in combination with MYC inhibitors and retinoids as a potential strategy for differentiation therapy in NB.

**Supplementary Information:**

The online version contains supplementary material available at 10.1038/s41598-026-38123-4.

## Introduction

Neuroblastoma (NB) is a neuroendocrine tumor of young children that is believed to originate from undifferentiated neural crest (NC) or NC-derived progenitor cells^1–3^. It is the most common extra cranial solid tumor, and accounts for approximately 15% of all cancer related deaths in children^1–3^. Clinically, NB is a heterogeneous tumor and NB patients are stratified into low, intermediate, and high-risk groups^4–6^. Low- and intermediate-risk patients have a favorable outcome with an event-free survival rate of 80–95%, while high-risk patients demonstrate < 50% event-free survival rate^7,8^. Current treatment of high-risk patients includes traditional intensive chemotherapy followed by surgical removal, radiation, myeloablation and autologous bone marrow transplantation, immunotherapy, as well as retinoid therapy^9–14^. Nevertheless, relapse and death due to refractory disease is common in high-risk patients. Thus, high-risk NB is considered a disease with unmet medical need.

Amplification of the *MYCN* gene occurs in ∼20% of all NB cases and about 40% of high-risk cases, and it is considered as marker of aggressive disease and poor survival^1–3^. Interestingly, 10% of high-risk tumors without *MYCN* amplification display elevated expression of MYC or the MYC pathway signature, suggesting a prominent, general role for the MYC family in aggressive NB forms^15–17^. The *MYC* family of oncogenes, which includes *MYC (*c-*MYC)*, *MYCN*, and *MYCL*, encode transcription factors of the basic region/helix-loop-helix/leucine zipper (bHLHZip) family and has been estimated to directly control around ∼15% of all genes in the genome, which impact almost all cellular processes such as proliferation, differentiation, DNA damage, metabolism, cell cycle, cell death, senescence, and self-renewal among others^18–20^. For many decades, the MYC family of oncoproteins have been described as ´´undruggable´´ due to their disordered protein structure and the lack of effective binding pockets or enzymatic activity. Therefore, targeting MYC activity in cancer has been focused on inhibiting MYC gene transcription, MYC interactions with its partner MAX, or targeting other MYC interacting proteins and pathways that are essential for MYC function^21–26^. Such efforts have led to the discovery of several small molecules inhibiting MYC/MYCN: MAX interactions^26–29^ and of Omomyc, a dominant-negative mini-protein that disrupts MYC-MAX interactions, which in turn abolishes MYC activity in cells^30,31^. This led to the clinical development of OMO-103, which showed a favorable safety profile, evidence of target engagement, and promising disease stabilization in a Phase I trial^32^, with Phase II studies in MYC-driven cancers currently ongoing. Nevertheless, it is important to develop several alternative strategies to combat MYC/MYCN-driven cancers including NB.

In NB, genome-wide CRISPR-Cas9 and drug screen studies have identified target genes/proteins that upon their inhibition imposed synthetic lethal anti-tumor activity that potentially could be utilized as a novel therapeutic approach^33–40^. Cyclin dependent kinase 2 (CDK2), which is a protein kinase involved in cell cycle progression, has been identified as a synthetic lethal target in NB cells with *MYCN*-amplification^41^. Inhibitors targeting CDK2 as well as other CDKs have been suggested as potential therapeutic agents in NB, especially in high-risk tumors with *MYCN*-amplification^42–45^. CDK2-mediated phosphorylation modulates the functions of a plethora of protein substrates involved in wide range of cellular pathways including cell cycle, DNA repair mechanisms, gene transcription, chromatin organization, RNA splicing, protein stability and degradation^46,47^. Notably, CDK2 has been shown to phosphorylate the MYC protein at the serine-62 residue^48^, and recent reports have shown a promising therapeutic advantage of CDK2 inhibitors targeting MYC-protein phosphorylation and stability in MYC-driven tumor models^49,50^.

How CDK2 impacts NB development, especially tumors with *MYCN-*amplification, is not fully understood. In this study, we uncovered that high CDK2 expression is a marker of advanced and aggressive NB as well as indicative of poor survival. We demonstrate for the first time a previously undefined function of CDK2 as a negative regulator of neuronal differentiation in human NB cell lines. Our experiments show that CDK2 knockdown or pharmacological inhibition induces neuronal differentiation and suppresses the MYC pathway. We also reveal a crosstalk between MYCN and CDK2, where MYCN controls CDK2 expression by directly targeting the CDK2 gene. Further, our data suggests that inhibition of CDK2 combined with MYC inhibitors or ATRA enhances neuronal differentiation and is potentially a new promising therapeutic strategy in NB.

## Results

### High CDK2 expression in neuroblastoma correlates with advanced disease stage, high-risk, *MYCN-*amplification and poor prognosis

**Fig. 1:**
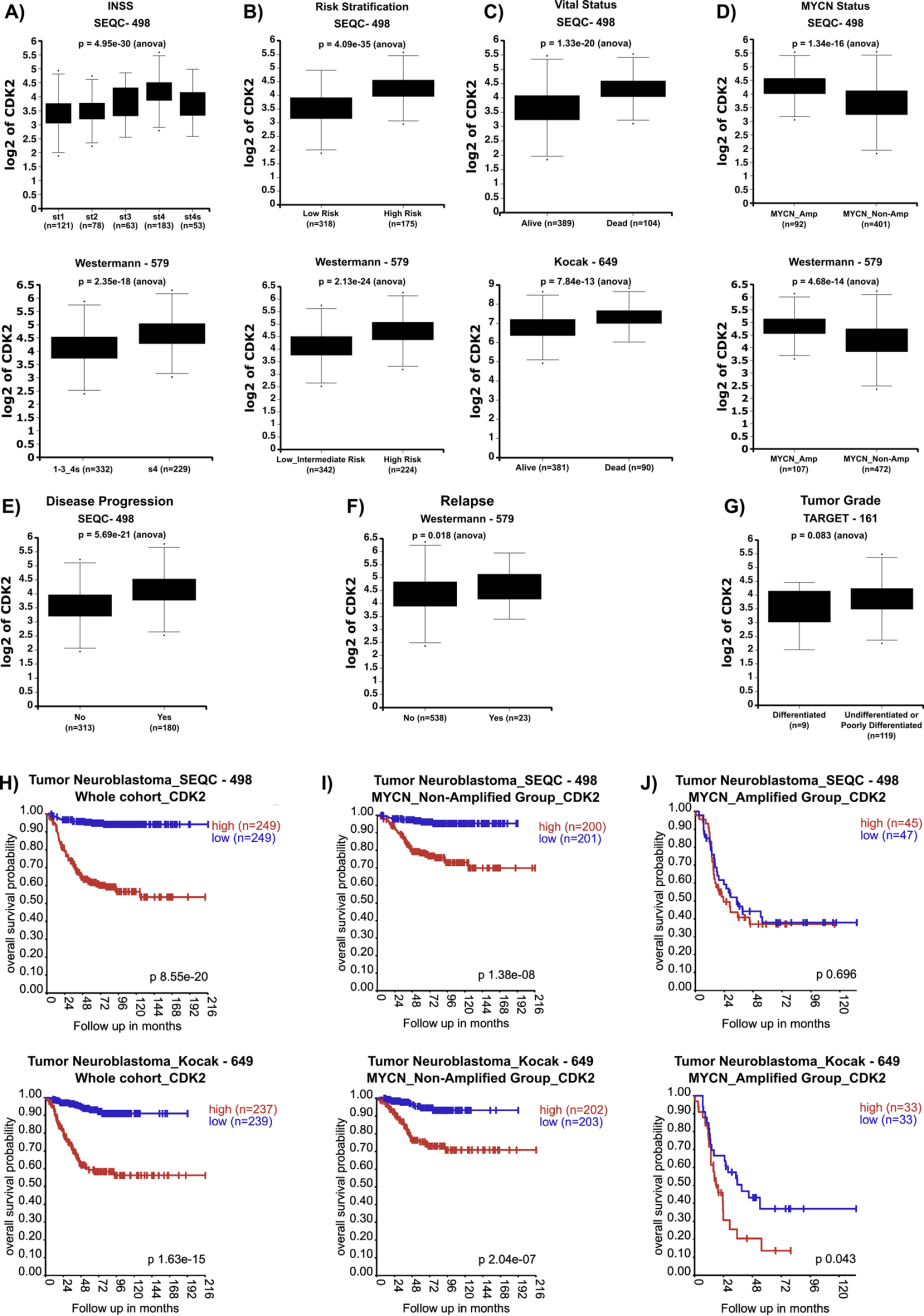
High CDK2 expression levels correlate with advanced and high-risk neuroblastoma, *MYCN*-amplification, and poor prognosis. Log_2_ of CDK2 mRNA levels in different NB stages as defined by The International Neuroblastoma Staging System (INSS) (**A**), NB risk stratification (**B**), in alive and dead NB patients (**C**), *MYCN*-amplification status (**D**), disease progression (**E**), Relapse (**F**), and differentiation state (**G**). Kaplan–Meier-analysis of overall survival of NB patients based on CDK2 expression in the SEQC-498 and KOCAK-649 whole cohort (**H**), *MYCN*-non-amplified subgroup (**I**), *MYCN*-amplified subgroup (**J**). Statistical significance was calculated using R2-Genomics built-in one-way ANOVA for correlation of CDK2 mRNA levels with clinical parameters, and Bonferroni correction of raw *p*-values for survival analysis.

To shed light on the impact of CDK2 in NB, we first sought to uncover the clinical and prognostic relevance of CDK2 expression in NB. Thus, we performed in-silico analysis correlating *CDK2* mRNA levels to clinical parameters in four publicly available NB cohort studies; the SEQC-498, Westermann-579, Kocak-649, and TARGET-161 deposited at the R2: Genomics Analysis and Visualization Platform (http://r2.amc.nl). As shown in Fig. 1A and supplementary Figure S1A, *CDK2* expression was highest in stage 4 (st4) NB when compared to other stages st1, st2, st3 and st4s in all datasets (Fig. 1A, Supplementary Figure S1A). Further, in the SEQC-498 and Westermann-579 cohorts in which NB patients were stratified into risk groups, we detected significantly higher *CDK2* mRNA levels in patients with high-risk disease (Fig. 1B). Moreover, *CDK2* mRNA levels were significantly higher in deceased NB patients when compared to alive patients as defined in the SEQC-498 and Kocak-649 cohorts (Fig. 1C) and the TARGET-161 cohort (Supplementary Figure S1B). With respect to *MYCN*-amplification status, *CDK2* mRNA levels were higher in *MYCN*-amplified NB in the SEQC-498 and Westermann-579 cohort studies (Fig. 1D) and the Kocak-649 cohort (Supplementary Figure S1C). Furthermore, we found *CDK2* mRNA levels to be significantly higher in progressive NB as classified in the SEQC-498 cohort (Fig. 1E), and in relapsed NB in the Westermann-579 cohort (Fig. 1F). Notably, *CDK2* mRNA levels were slightly higher in undifferentiated/poorly differentiated NB when compared to differentiated NB as defined in the TARGET-161 cohort (Fig. 1G). We further investigated the prognostic value of *CDK2* expression on the survival of NB patients in the SEQC-498 and Kocak-649 NB cohorts. High *CDK2* expression was indicative of poor overall survival of NB patients in both cohorts (Fig. 1H), as well as poor event-free survival in the SEQC-498 (Supplementary Figure S1D) and the Kocak-649 (Supplementary Figure S1E) NB cohorts. When dividing NB patients based on *MYCN*-amplification status, high *CDK2* expression was indicative of poor survival in the *MYCN*-non-amplified group in both datasets (Fig. 1I), while in the *MYCN*-amplified group high *CDK2* expression correlated significantly with poor survival in the Kocak-649 cohort but not the SEQC-498 cohort (Fig. 1J). Collectively, these results clearly indicate that high *CDK2* expression is indicative of advanced and aggressive tumors and marker of poor prognosis in NB.

### High CDK2 expression is indicative of proliferative and undifferentiated neuroblastoma 

**Fig. 2:**
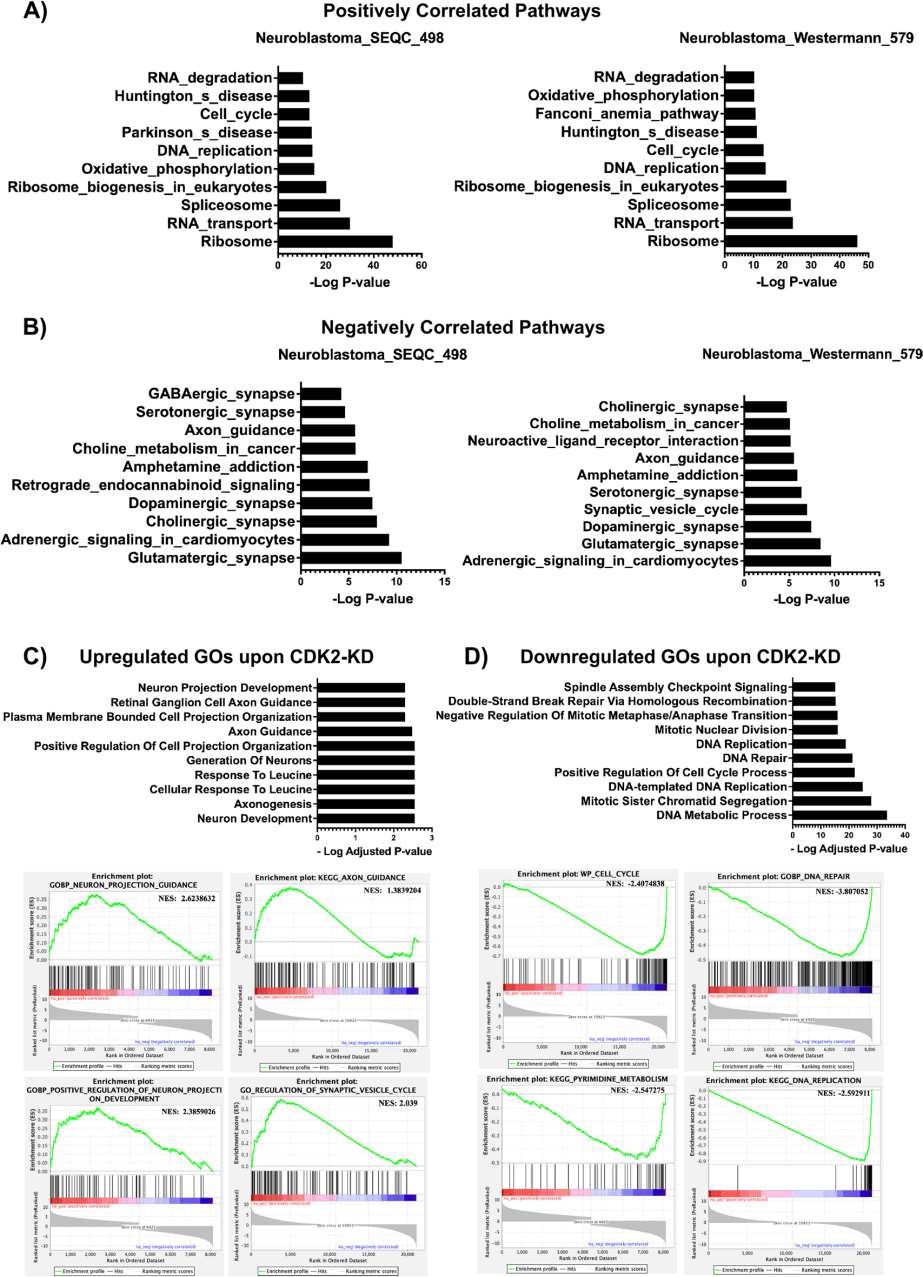
High CDK2 expression levels correlate with cell proliferation and differentiation block in NB. KEGGpathway analysis of genes whose expression positively (**A**) and negatively (**B**) correlated with CDK2 in the SEQC-498 and Westermann-579 NB cohort studies. KEGG analysis was performed using the R2-genomics platform. (**C-D**) Gene ontology analysis and GSEA of upregulated genes (**C**), and down-regulated genes (**D**) following CDK2 knockdown in the *MYCN*-amplified IMR32 cell line. Gene ontology and GSEA of differentially regulated genes were performed using Gene Set Enrichment Analysis (Broad institute, GSEA version 4.2.2) and Enrichr: a comprehensive gene set enrichment analysis online server.

To investigate the biological relevance of *CDK2* expression in NB, we performed pathway analysis on RNA gene expression datasets of the SEQC-498, Westermann-579, Kocak-649, and TARGET-161 cohort studies using the R2: Genomics Analysis and Visualization Platform. KEGG pathway analysis^51^ revealed that *CDK2* expression positively correlated with the expression of genes related to cell cycle as expected, but also ribosome biogenesis, RNA transport, DNA replication, DNA repair mechanisms, RNA splicing and nucleotide metabolism in all datasets (Fig. 2A, Supplementary Figure S2A, and Table S1). On the other hand, *CDK2* expression negatively correlated with genes involved in neuronal activity, neuronal synapses, axon guidance, neuronal ligands and receptors, cell adhesion, immune and inflammatory pathways (Fig. 2B, Supplementary Figure S2B, and Table S1). Figure 2B and supplementary Figure S2B show the top 10 neuronal related pathways negatively correlated with *CDK2* expression, while the complete list of pathways is found in Table S1. We further analyzed NB single cell gene expression datasets available at NB atlas (https://single-cell.be/nbatlas/)^52^ to investigate *CDK2* expression in different tumor cell subsets of NB. We detected *CDK2* expression mainly in two cellular clusters: cluster 2: Cycling-S and cluster7: MYCN (Supplementary Figure S2C). The partial overlap between *CDK2* and *MYCN* expression in the tumor cell clusters indicates that *CDK2* is expressed only in a subset of *MYCN*-amplified/expressing NBs (Supplementary Figure S2C). There was no correlation between *MYC* and *CDK2* expression in any of the clusters (Supplementary Figure S2C), as well as in the four publicly available NB cohort studies analyzed in this study (data not shown). Notably, differentiation clusters C0 and C3 had very low expression of *CDK2* (Supplementary Figure S2C). The inverse correlation between *CDK2* expression and neuronal differentiation in human NB has not been investigated so far. To further corroborate this finding, we analyzed the publicly available gene expression dataset (GSE16480) with gene expression analysis in response to 72 h doxycycline inducible knockdown of CDK2 (CDK2-KD) in the human *MYCN*-amplified cell line IMR32^41^. Using gene set enrichment analysis (GSEA), we found that CDK2-KD resulted in the upregulation of genes related to neuronal development, axongenesis, generation of neurons, axon guidance, and response to leucine (Fig. 2C), while downregulated genes involved in cell cycle, DNA metabolic pathways, DNA repair, DNA replication, and chromosome segregation (Fig. 2D). Collectively, gene expression analysis of bulk and single cell RNA-seq data of primary NB tumors, as well as of the IMR32 cell line suggests CDK2 as marker of proliferative NB with suppressed neuronal differentiation capacity.

### CDK2 depletion induces neuronal differentiation and cell death in *MYCN*-amplified neuroblastoma

**Fig. 3:**
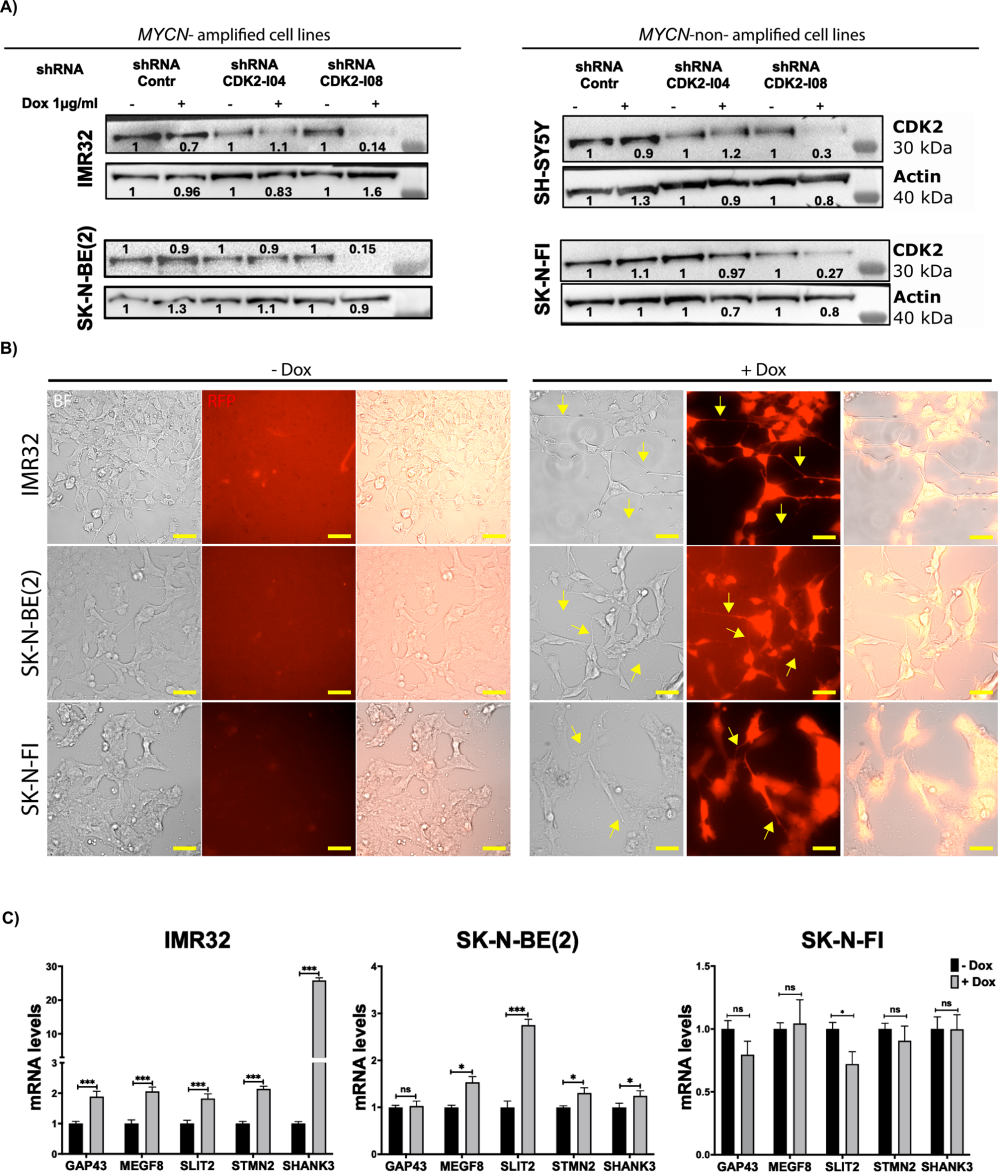
CDK2 knockdown induces neuronal differentiation and cell death in *MYCN*-amplified NB cell lines. (**A**) Western blot analysis of CDK2 protein levels in two *MYCN*-amplified cells lines IMR32 and SK-N-BE(2), and two *MYCN*-non-amplified cell lines SK-N-FI and SH-SY5Y transduced with doxycycline inducible non-silencing shRNA-control or two shRNA against CDK2; shRNA-I04 and shRNA-I08. Cells were treated with doxycycline of 1 µg/ml for 3 days. Actin was used as loading control for western blots. (**B**) bright field and red fluorescent protein (RFP) images to analyze neurite outgrowth in shRNA-CDK2-I08 expressing cell lines IMR32, SK-N-BE(2), and SK-N-FI treated with 1 µg/ml doxycycline for 6 days. Scale bars represent 50 μm. (**C**) qRT-qPCR analysis of neuronal differentiation markers in shRNA-CDK2-I08 transduced cell lines IMR32, SK-N-BE(2), and SK-N-FI treated with 1 µg/ml doxycycline for 6 days. Mock H_2_O treatment was used as control and GAPDH was used as housekeeping gene. Error bars represent the standard deviation of three independent biological experiments. The p-value was calculated using two-tailed, unpaired Student t-test in GraphPad Prism. * p-value ≤ 0.05; ** p-value ≤ 0.01; *** p-value ≤ 0.001.

The in-silico analysis presented above indicated a novel role for CDK2 in neuronal differentiation, which prompted us to examine if CDK2 regulates neuronal differentiation in human NB cells. To this end, we generated human NB cell lines with stable expression of doxycycline-inducible red fluorescent protein (RFP)-tagged shRNA against CDK2. The *MYCN*-amplified cell lines IMR32 and SK-N-BE(2) and the *MYCN*-non-amplified cell lines SK-N-FI and SH-SY5Y were transduced with two CDK2 targeting shRNAs, I04 and I08, or non-silencing shRNA-control. As shown in Fig. 3A, doxycycline treatment for 72 h reduced CDK2 protein levels in all NB cell lines transduced with CDK2-shRNA-I08, but not in cells transduced with non-silencing shRNA-control (Fig. 3A). The efficiency of CDK2-KD by shRNA-I04 was less than that of shRNA-I08 and varied between cell lines (Fig. 3A). Thus, we decided to continue our studies using cell lines transduced with shRNA-CDK2-I08 and shRNA-control. Then, we performed a 6-day differentiation assay in cells expressing CDK2-shRNA-I08 and non-silencing shRNA-control and monitored the induction of neurite outgrowth utilizing the RFP-tag (Fig. 3B and Supplementary Figure S3A). shRNA-mediated KD of CDK2 induced neurite extensions in *MYCN*-amplified IMR32 and SK-N-BE(2) cells, as well as in the *MYCN*-non-amplified SK-N-FI (Fig. 3B) and SH-SY5Y (Supplementary Figure S3A and 3B) cells. Induction of non-silencing shRNA-control did not result in neurite outgrowth in any of the cell lines (Supplementary Figure S3A). We further assessed neuronal differentiation following CDK2-KD in NB cell lines transduced with shRNA-I08 by analyzing the expression of mRNA levels of genes involved neuronal differentiation, axongenesis, neuronal projection, and synapse formation; *GAP43*, *MEGF8*, *SLIT2*, *STMN2*, and *SHANK3* identified in our differential gene expression analysis of the IMR32 cell line publicly available gene expression dataset (GSE16480)^41^. Notably, doxycycline-mediated knockdown of CDK2 upregulated the expression of these genes in *MYCN-*amplified IMR32 and SK-N-BE(2) cells (Fig. 3C), but not in *MYCN-*non*-*amplified SK-N-FI (Fig. 3C) and SH-SY5Y cells (Supplementary Figure S3C), where expression of some of these genes was even decreased (Fig. 3C and supplementary Figure S3C). Previously, Molenaar et al. 2009 have demonstrated that depletion of CDK2 induces cell death in *MYCN*-amplified cell lines^41^. Thus, we analyzed the impact of CDK2-KD on cell death by assessing the levels of cleaved PARP by western blot. We found that siRNA-mediated KD of CDK2 triggered PARP cleavage in IMR32 and SK-N-BE(2) cells, indicative of induced apoptosis (Supplementary Figure S3D). Collectively, these findings indicate that CDK2 depletion induces a combination of neuronal differentiation and cell death in *MYCN*-amplified NB cell lines. Morphological signs of differentiation were observed also in the *MYCN-*non*-*amplified cell lines, but induction of differentiation-related genes was less pronounced in these cells.

### Pharmacological Inhibition of CDK2 induces neuronal differentiation in neuroblastoma cell lines

**Fig. 4:**
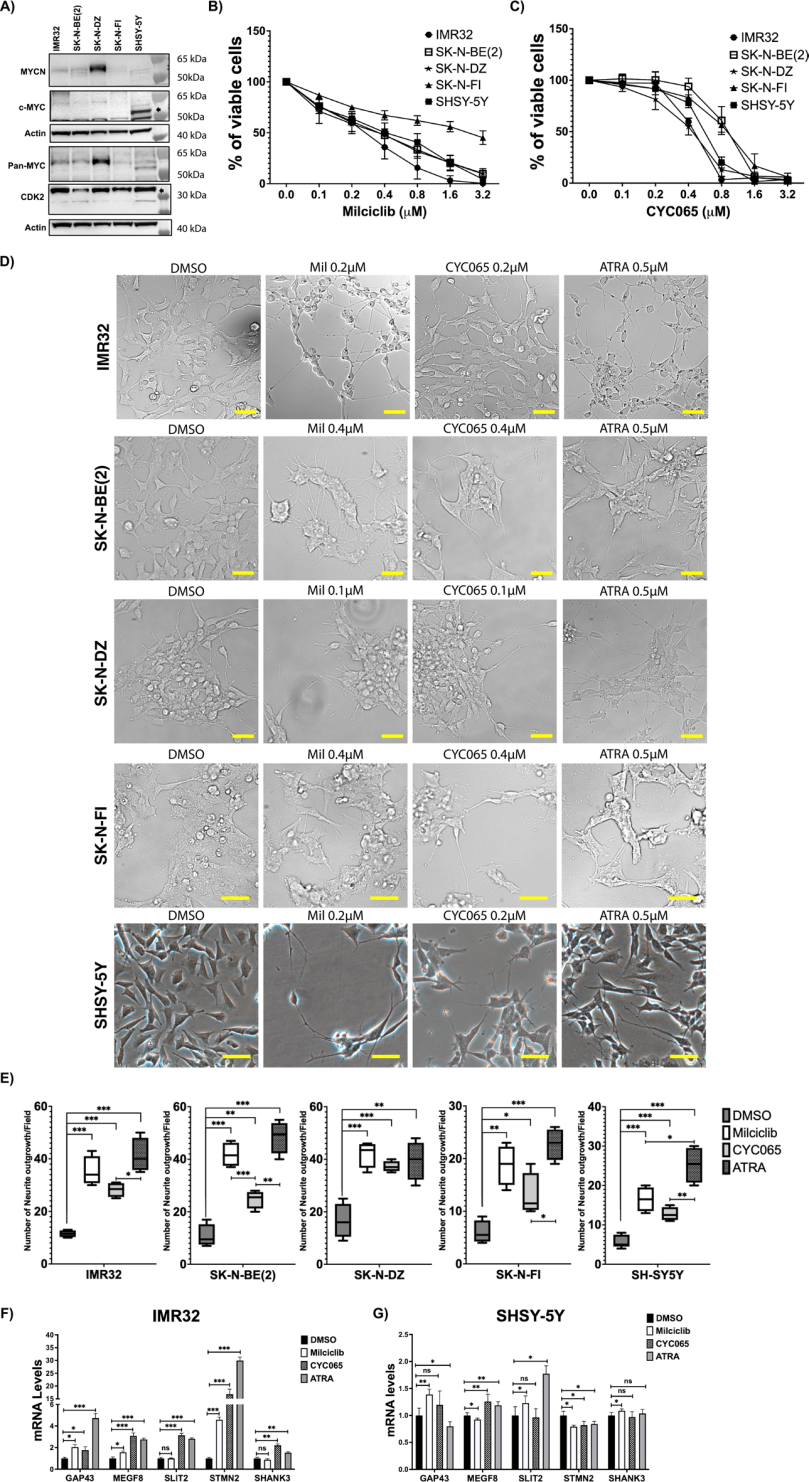
CDK2 pharmacological inhibition induces neuronal differentiation in NB cell lines. (**A**) Western blot analysis of MYCN, MYC, Pan-MYC, CDK2, and actin protein levels in a set of human neuroblastoma cell lines used in this study. IMR32, SK-N-DZ, and SK-N-BE(2) represent *MYCN*-amplified cell lines. SK-N-FI and SH-SY5Y represent *MYCN*-non-amplified neuroblastoma cell lines. Actin was used as loading control. (**B-C**) Resazurin-based cell viability assay of neuroblastoma cell lines treated with the CDK2 inhibitors Milciclib (**B**) and CYC065 (**C**) for 72 h. (**D**) Bright field images analyzing neurite outgrowth in IMR32, SK-N-BE(2), SK-N-DZ, SK-N-FI, and SH-SY5Y cells treated for 6 days with the indicated concentrations of milciclib, CYC065, and ATRA. DMSO was used as control treatment. Scale bars represent 50 μm. (**E**) Quantification of neurite outgrowth number in all cell lines as indicated in Fig. 4D. Number of neurite outgrowth was manually counted using ImageJ software. Bars represent the median number of neurite extensions per field of three independent biological experiments. (**F-G**) qRT-qPCR analysis of neuronal differentiation markers in IMR32 (**F**) and SH-SY5Y (**G**) treated with 0.2 µM of milciclib, 0.2 µM of CYC065, or 0.5 µM of ATRA for 3 days. DMSO was used as control treatment and GAPDH was used as housekeeping gene. Error bars represent the standard deviation of three independent biological experiments. The p-value was calculated using two-tailed, unpaired Student t-test in GraphPad Prism. * p-value ≤ 0.05; ** p-value ≤ 0.01; *** p-value ≤ 0.001.

Having shown that CKD2-KD promotes neuronal differentiation in NB cell lines, we sought to evaluate whether pharmacological inhibition of CDK2 has similar effects in these cell lines. For this purpose, we utilized the CDK2/TRKA inhibitor milciclib and the CDK2/9 inhibitor CYC065 and first evaluated their impact on the viability of a set of NB cell lines with and without *MYCN*-amplification. As assessed by western blotting, the *MYCN*-amplified cell lines IMR32, SK-N-BE(2) and SK-N-DZ expressed MYCN protein, but not MYC (Fig. 4A). Among the two *MYCN*-non-amplified cell lines, SK-N-FI cells expressed low levels of MYCN, while SH-SY5Y cells expressed MYC protein (Fig. 4A). Results of pan-MYC antibody staining confirmed that the *MYCN*-amplified cell lines had in general higher total MYC-family protein levels compared to the *MYCN*-non-amplified cell lines (Fig. 4A). All cell lines had comparable levels of the CDK2 protein (Fig. 4A). Results of resazurin cell viability assay demonstrated that 72 h treatment with milciclib or CYC065 decreased cell viability of all cell lines in a dose dependent manner irrespective of *MYCN*-amplification status (Fig. 4B and C). To assess the effect of CDK2 pharmacological inhibition on neuronal differentiation of NB cell lines, we treated the cell lines with low doses of milciclib and CYC065 for 6-days, followed by microscopic assessment of neurite extensions. Both milciclib and CYC065 induced significant increase in the number of neurite extensions/outgrowths in all five cell lines (Fig. 4D and E), although slightly less than the positive control all-t*rans*-retinoic acid (ATRA), except in SK-N-DZ, where the effects of milciclib, CYC065, and ATRA were similar (Fig. 4E). qRT-PCR analysis of *GAP43*, *MEGF8*, *SLIT2*, *STMN2*, and *SHANK3* mRNA levels indicated a significant increase in the expression of most markers in *MYCN*-amplified IMR32 cells following 72 h treatment with milciclib and CYC065 (Fig. 4F). This was less consistent in *MYCN*-non-amplified SH-SY5Y cells, with essentially all the five markers showing variable responses between no change, increase, or decrease in response to the treatments (Fig. 4G).

**Fig. 5:**
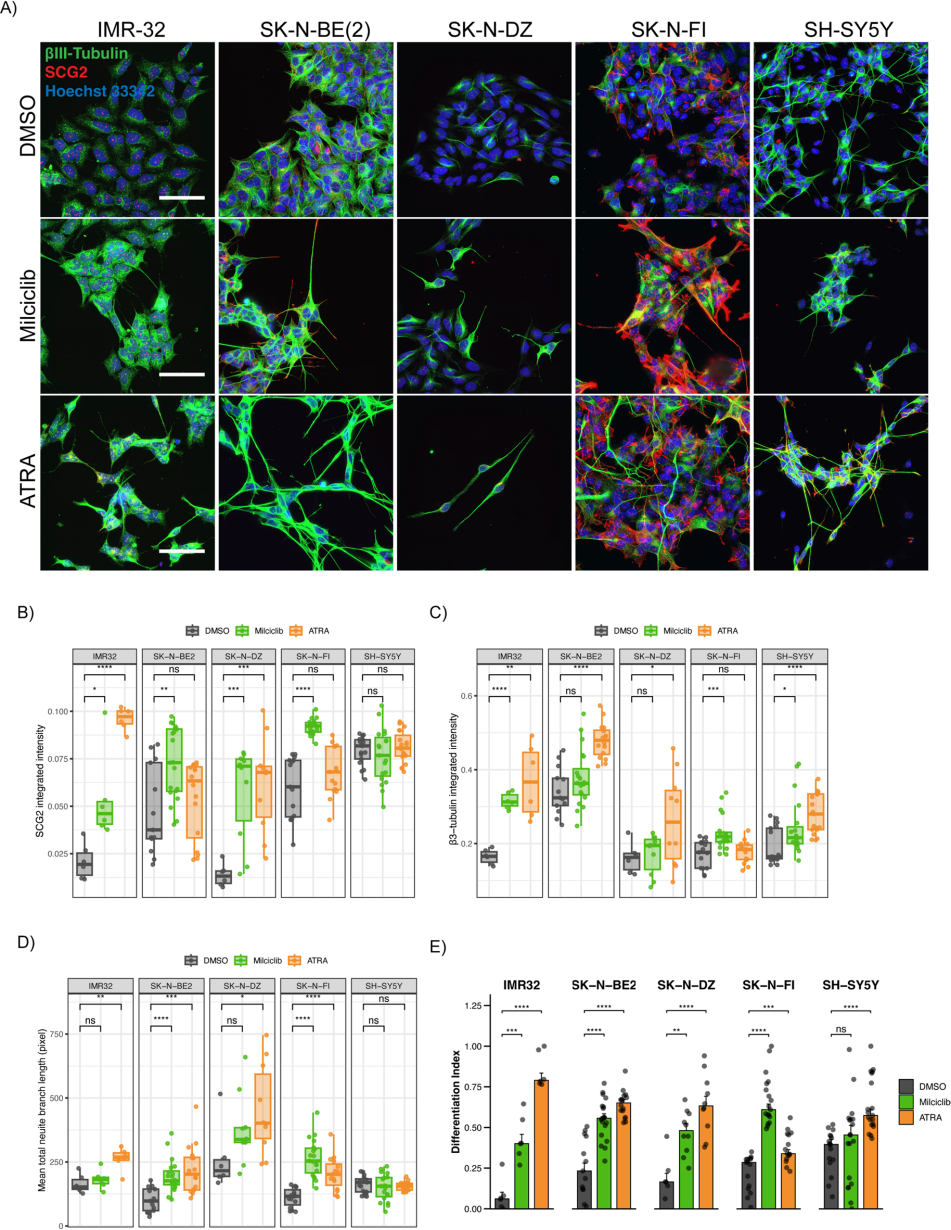
CDK2 pharmacological inhibition induces neuronal differentiation in NB cell lines. (**A**) Immunofluorescent staining of SCG2 (Red), β3-Tub (Green), and DNA (Hoechst 33342) in human neuroblastoma cell lines. IMR32, SK-N-DZ, and SK-N-BE(2) represent *MYCN*-amplified cell lines, SK-N-FI and SH-SY5Y represent *MYCN*-non-amplified neuroblastoma cell lines. The cells were exposed to 6-days treatment with milciclib and ATRA as indicated in Fig. 4. (**B**) Quantification of SCG2 integrated signal intensity in Fig. 5A. (**C**) Quantification of integrated β3-Tub signal intensity in Fig. 5A. (**D**) Quantification of mean total of neurite length in Fig. 5A. (**E**) Differentiation index of NB cell lines combining the SCG2 and β3-Tub intensities as well as neurite length in Fig. 5A. DMSO was used as a control treatment. The p-value was considered significant at *p* < 0.05. (*N* ≥ 3, *n* ≥ 6, median ± SEM).

To further assess the effects of CDK2 inhibition on the neuronal phenotype of NB cells, we treated all cell lines with milciclib, ATRA or DMSO control for 6 days then assessed by immunofluorescence analysis the levels of secretogranin II (SCG2) and beta 3 tubulin (β3-Tub), which are two well established markers of neuronal differentiation^53,54^, as well as the length of neurite outgrowths (Fig. 5A). Milciclib treatment resulted in significant increase of SCG2 intensity in all cell lines except in SH-SY5Y, which had high SCG2 intensity already in the control-treated cells (Fig. 5A and B). Also the β3-Tub intensity was significantly higher in milciclib-treated IMR32, SK-N-FI, and SH-SY5Y cells when compared to DMSO control treatment (Fig. 5A and C), Further, milciclib significantly increased neurite length in SK-N-BE(2), SK-N-DZ, and SK-N-FI, but not in IMR32 and SH-SY5Y cells (Fig. 5A and D). As a comparison, treatment with the ATRA positive control significantly increased SCG2 intensity in IMR32 and SK-N-DZ, but not in SK-N-BE(2), SK-N-FI, and SH-SY5Y cells (Fig. 5A and B) and it significantly increased β3-Tub intensity in all cell lines except SK-N-FI (Fig. 5A and C). ATRA also significantly increased neurite length in all the cell lines except SH-SY5Y (Fig. 5A and D). To define a differentiation index for each cell line, we combined all the parameters i.e., SCG2 and β3-Tub intensities with neurite length (see Materials & Methods), and found that milciclib treatment resulted in significantly higher differentiation index in all cell lines when compared to DMSO control except in SH-SY5Y cell line (Fig. 5E), while ATRA increased the differentiation index of all cell lines (Fig. 5E). These data indicate that pharmacological inhibition of CDK2 induces neuronal differentiation in NB cell lines to a level comparable to ATRA treatment.

### *CDK2* is a MYCN target gene and regulator of the MYC pathway

**Fig. 6:**
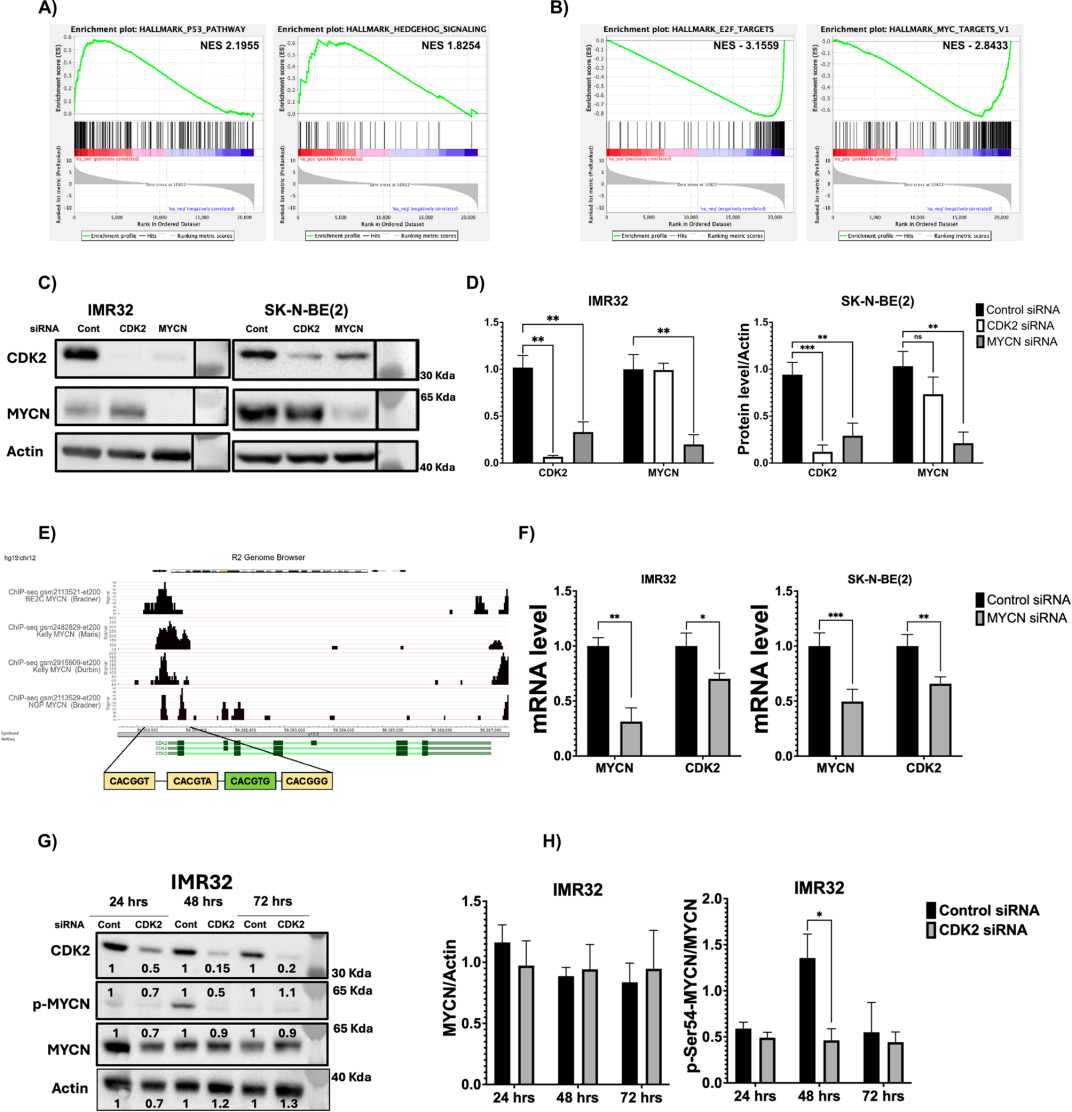
*CDK2* is a MYCN target gene that modulate the MYC pathway in *MYCN*-amplified neuroblastoma. (**A-B**) GSEA of cancer hallmarks of differentially upregulated (**A**) and downregulated (**B**) genes following CDK2 knockdown in the *MYCN*-amplified IMR32 cell line. (**C**) Westen blot analysis of CDK2 and MYCN protein levels in the *MYCN*-amplified cell lines IMR32 and SK-N-BE(2) transfected with control siRNA, CDK2 siRNA, or MYCN siRNA for 3 days. (**D**) Quantification of CDK2 and MYCN protein levels in Fig. 4C. (**E**) Analysis of MYCN ChIP-Seq data around CDK2 gene transcription start site in the *MYCN*-amplified SK-N-BE(2)C, KELLY, and NGP cell lines. Yellow labeled boxes indicate non-canonical E-boxes, while green labeled box indicates canonical E-boxes. (**F**) Quantification of *MYCN* and *CDK2* mRNA levels following siRNA-mediated MYCN knockdown in IMR32 and SK-N-BE(2) cells for 72 h. (**G**) Western blot analysis of CDK2, phosphorylated-Ser54-MYCN, and total MYCN protein levels in *MYCN*-amplified IMR32 cells transfected with control or CDK2 siRNA for 24, 48, and 72 h. (**H**) Quantification of total MYCN protein levels and the ratio of phosphorylated-Ser54-MYCN/MYCN at the time points shown in Fig. 4E. Actin was used as loading control in all western blots. In all blots, the last lane represents the molecular weight protein ladder. In Fig. 4C ladder lane was aligned next to the corresponding lanes after removal of an in between irrelevant lane (for clarification see supplementary uncropped western blots file). Error bars represent the standard deviation of three independent biological experiments. The p-value was calculated using two-tailed, unpaired Student t-test in GraphPad Prim. * p-value ≤ 0.05; ** p-value ≤ 0.01; *** p-value ≤ 0.001.

To get insights into how CDK2 affects neuronal differentiation in NB, we further analyzed differential gene expression in the publicly available gene expression array (GSE16480) in response to 72 h dox-induced CDK2-KD in the IMR32 cell line^41^. We found that CDK2-KD resulted in upregulation of TP53 target genes and of the sonic hedgehog pathway (Fig. 6A) but reduced the expression of E2F and MYC target genes (Fig. 6B). MYCN has been described as a major factor blocking neuronal differentiation in NB^55,56^. Therefore, we focused our subsequent analyses on the potential functional crosstalk between CDK2 and MYCN. To this end, we first transfected IMR32 and SK-N-BE(2) cells with siRNAs targeting CDK2 or MYCN or with control siRNA. We found that siRNA-mediated-KD of MYCN dampened CDK2 protein levels in both IMR32 and SK-N-BE(2) cells (Fig. 6C and D). KD of CDK2 did not reduce MYCN protein levels in IMR32 cells, but led to a slight but not significant decrease in MYCN levels SK-N-BE(2) cells (Fig. 6C and D). To further investigate if MYCN regulates CDK2 expression in NB, we utilized the TET21N NB cell line with doxycycline-regulatable MYCN protein expression^57^. Markedly, doxycycline-induced downregulation of MYCN led to reduced CDK2 protein level also in these cells (Supplementary Figure S4A). To uncover whether MYCN directly regulates *CDK2* transcription, we analyzed MYCN ChIP-Seq data available at the R2-Genomic Platform and detected an enrichment of MYCN ChIP-Seq peaks around the *CDK2* transcription start-site in three *MYCN*-amplified cell lines, SK-N-BE(2)C, KELLY and NGP (Fig. 6E). Notably, the DNA sequence under MYCN ChIP-Seq peaks contained four E-boxes: one canonical (CACGTG), and three non-canonical E-boxes (Fig. 6E). To further validate if *CDK2* is a MYCN target gene in NB, we analyzed *CDK2* mRNA levels following MYCN KD in IMR32 and SK-N-BE(2) cells. The siRNA-mediated KD of MYCN reduced *CKD2* mRNA levels in both cell lines (Fig. 6F). Collectively, these findings establish *CDK2* as target gene of MYCN in neuroblastoma.

Previously, CDK2 was shown to affect MYC phosphorylation at Ser62^48^, a site reported to regulate MYC protein activity and stability^58^. This prompted us to study the impact of CDK2-depletion on Ser54 MYCN phosphorylation (the equivalent to MYC-Ser62 phosphorylation) in *MYCN*-amplified IMR32 and SK-N-BE(2) cells. We performed a time course experiment over 72 h and found that Ser54-MYCN phosphorylation fluctuated in control-siRNA-treated cells with a peak reproducibly occurring at 48 hours post treatment (Fig. 6G and H and Supplementary Figure S4B). In contrast, Ser54-MYCN phosphorylation was low in CDK2 siRNA-treated cells at all time points, suggesting that CDK2 affects MYCN phosphorylation in these cells. Total MYCN protein levels were not significantly affected at any time point, in agreement with Fig. 6C and D. In SK-N-BE(2) cells, Ser54-MYCN phosphorylation was somewhat reduced in parallel with total MYCN levels at 48 hours post CDK2-KD, and therefore the phospho-MYCN/MYCN ratio was unchanged (Supplementary Figure S4B). The effects of CDK2-KD on MYCN protein levels were also recapitulated in IMR32 and SK-N-BE(2) cells transduced with doxycycline inducible CDK2-shRNA I08 expression vector, where MYCN protein levels were slightly affected upon CDK2 knockdown by doxycycline treatment for 72 h, but these changes did not reach significance (Supplementary Figure S4C). Similarly, we did not notice significant changes in MYC protein levels following doxycycline inducible CDK2-KD in SH-SY5Y cells (Supplementary Figure S4D). These data establish *CDK2* as MYCN target in NB that modulates the MYC pathway, while the impact of CDK2 on MYCN phosphorylation and stability in NB cells was less clear and needs further investigation.

### Combinations of CDK2 and MYC inhibitors trigger synergistic or additive anti-neuroblastoma effects and enhance neuronal differentiation

Considering the crosstalk between CDK2 and the MYC pathway, we also evaluated the effect of two MYC-inhibitory compounds; the BET-bromodomain inhibitor JQ1 reported to dampen MYC/MYCN transcription^59^ and MYCMI-7, which disrupts both MYC: MAX^29^ and MYCN: MAX interactions^60^. The treatment with JQ1 for 72 h resulted in a substantial dose dependent effect on cell viability in IMR32, SK-N-BE(2), SK-N-DZ, and SH-SY5Y cells, while showing less effect in SK-N-F1 cells (Supplementary Figure S5A). Additionally, MYCMI-7 reduced the viability of all *MYCN*-amplified cell lines except SK-N-BE(2) but had weaker effects on the *MYCN*-non-amplified cell lines (Supplementary Figure S5B). Like the CDK2 inhibitors, JQ1 and MYCMI-7 induced neurite extension in both *MYCN*-amplified and *MYCN*-non-amplified cell lines (Supplementary Figure S5C and S5D). We next analyzed the impact of CDK2 and MYC/MYCN inhibitors on RB- and MYCN-phosphorylation, total RB, CDK2 and MYCN protein levels as well as signs of apoptosis in *MYCN*-amplified SK-N-DZ (Supplementary Figure S6A) and IMR32 (Supplementary Figure S6H) cells. Both milciclib and CYC065 CDK2 inhibitors reduced RB phosphorylation at Thr821, a CDK2 phosphorylation site, without affecting the total RB protein level in SK-N-DZ (Supplementary Figure S6A-D) and IMR32 cells (Supplementary Figure S6H-K) as expected, while among the MYC inhibitors, only JQ1 treatment reduced the phospho-Thr821/RB ratio in IMR32 cells (Supplementary Fig. 6H-K). Further, while the CDK2- and MYC/MYCN-inhibitors did not affect phosphorylation of MYCN Ser54 or the total level of MYCN in SK-N-DZ cells (Supplementary Figure S6A, E-G), they all reduced both Ser54-MYCN phosphorylation and total MYCN levels (therefore not affecting the phopho-Ser54-MYCN/MYCN ratio) in IMR32 cells (Supplementary Figure S6H, L-N). Moreover, milciclib and CYC065 reduced CDK2 protein levels to various degrees in SK-N-DZ and IMR32 cells (Supplementary Fig. 6 A and H), while MYCMI-7 reduced CDK2 protein levels only in IMR32 cells (Supplementary Fig. 6H). Regarding signs of cell death, we found that milciclib or CYC065 treatment induced PARP cleavage in SK-N-DZ (Supplementary Fig. 6 A) and IMR32 (Supplementary Fig. 6H) cells. MYCMI-7 treatment induced PARP cleavage in IMR32 cells (Supplementary Fig. 6H), while JQ1 treatment slightly increased PARP cleavage in SK-N-DZ cells (Supplementary Fig. 6 A). Collectively, these data demonstrate that single treatments with CDK2 and MYC/MYCN inhibitors induce neuronal differentiation in NB cell lines and causes cell death in *MYCN*-amplified cell lines.

The observation that both CDK2- and MYC/MYCN-inhibitors induced differentiation of NB cells, prompted us to evaluate the effects of combination treatments with CDK2 and MYC inhibitors on viability and differentiation of NB cells. We found that combination of milciclib and JQ1 further reduced the viability of *MYCN*-amplifed IMR32 and SK-N-BE(2) cells as well as non-*MYCN*-amplified SK-N-F1 and SHSY-5Y cells when compared to single drug treatments (Supplementary Figure S7A, C, E, and G). We next investigated whether these combinatorial effects were synergistic, additive, or antagonistic using Bliss-synergy score model, where a score of less than − 10 indicates antagonism; a score of −10 to 10 indicates additive effects; while synergy score larger than 10 suggests synergistic action^61^. Milciclib and JQ1 combinations demonstrated synergetic effects in SK-N-BE(2) and SK-N-FI cells and additive effects in IMR32 and SH-SY5Y cells, and thus did not seem to correlate with *MYCN* amplification status (Supplementary Figure S7A, C, E, and G). Notably, combination of milciclib and JQ1 further reduced MYCN protein levels in SK-N-BE(2) and IMR32 cells, and CDK2 protein level in IMR32 cells when compared to single agent treatment (Supplementary Figure S7I). We also evaluated the combination of milciclib and MYCMI-7 on the viability of NB cells utilizing the same four NB cell lines and found that milciclib and MYCMI-7 combinations had additive effects in all cases (Supplementary Figure S8A-H). Like milciclib and JQ1 combination treatment, combined treatment with milciclib and MYCMI-7 led to further reduction in MYCN and CDK2 protein levels when compared to single treatments in the *MYCN*-amplified IMR32 cells but not in *MYCN*-non-amplified SK-N-FI cells (Supplementary Figure S8I).

**Fig. 7:**
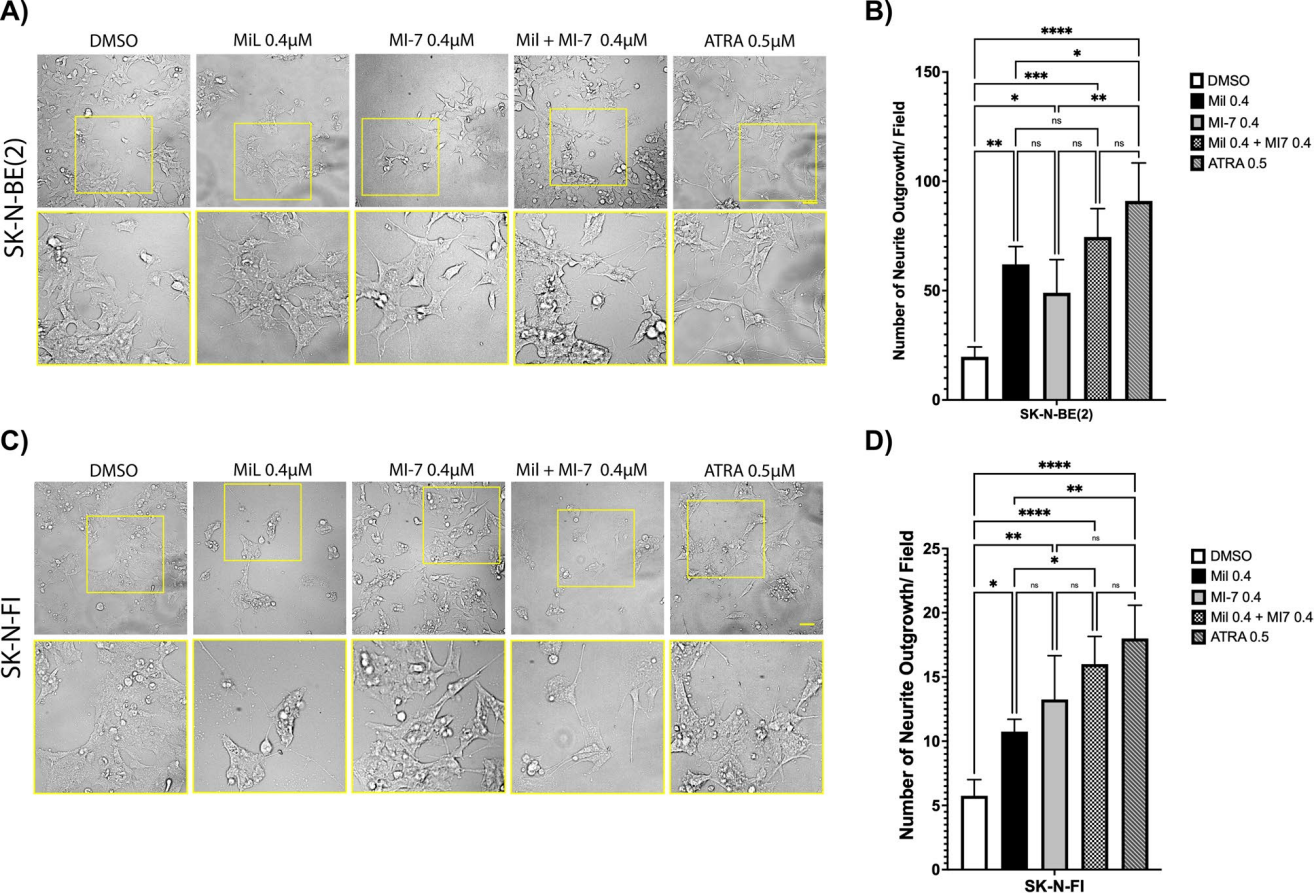
Combination of CDK2 and MYCN inhibitors enhances neuronal differentiation of NB cells. *MYCN*-amplified SK-N-BE(2) (**A-B**) and *MYCN*-non-amplified SK-N-FI (**C-D**) cells were treated with milciclib or MYCMI-7 in single and combinatorial settings as indicated for 6 days, after which cells were microscopically analyzed (**A** and **C**) for neurite outgrowths. DMSO was used as control treatment. (**B** and **D**) Quantification of neurite outgrowth was manually counted using ImageJ software. Bars represent the median number of neurite extensions per field of three independent biological experiments. Scale bars represent 50 μm. The p-value was calculated using one way-Anova for multiple comparisons in GraphPad Prism. * p-value ≤ 0.05; ** p-value ≤ 0.01; *** p-value ≤ 0.001, **** p-value ≤ 0.0001.

Next, we evaluated whether combination of the CDK2 and MYC/MYCN inhibitors milciclib and MYCMI-7, respectively, can impact neuronal differentiation. To this end, we utilized the *MYCN*-amplified SK-N-BE(2) and the *MYCN*-non-amplified SK-N-FI cell lines, both expressing the MYCN protein (Fig. 4A). As shown in Fig. 7, single drug treatment induced a significant increase in the number of neurite extensions in both cell lines when compared to DMSO control treatment (Fig. 7B and D). The combination of milciclib and MYCMI-7 further increased the number of neurite outgrowths in both SK-N-BE(2) and SK-N-FI cells although this was not statistically significant (Fig. 7B and D). These findings suggest that combination of CDK2 and MYC/N inhibition reduces cell viability/growth of NB cell lines and enhances neuronal differentiation.

### Combination of milciclib and ATRA enhances neuronal differentiation of neuroblastoma cells

**Fig. 8:**
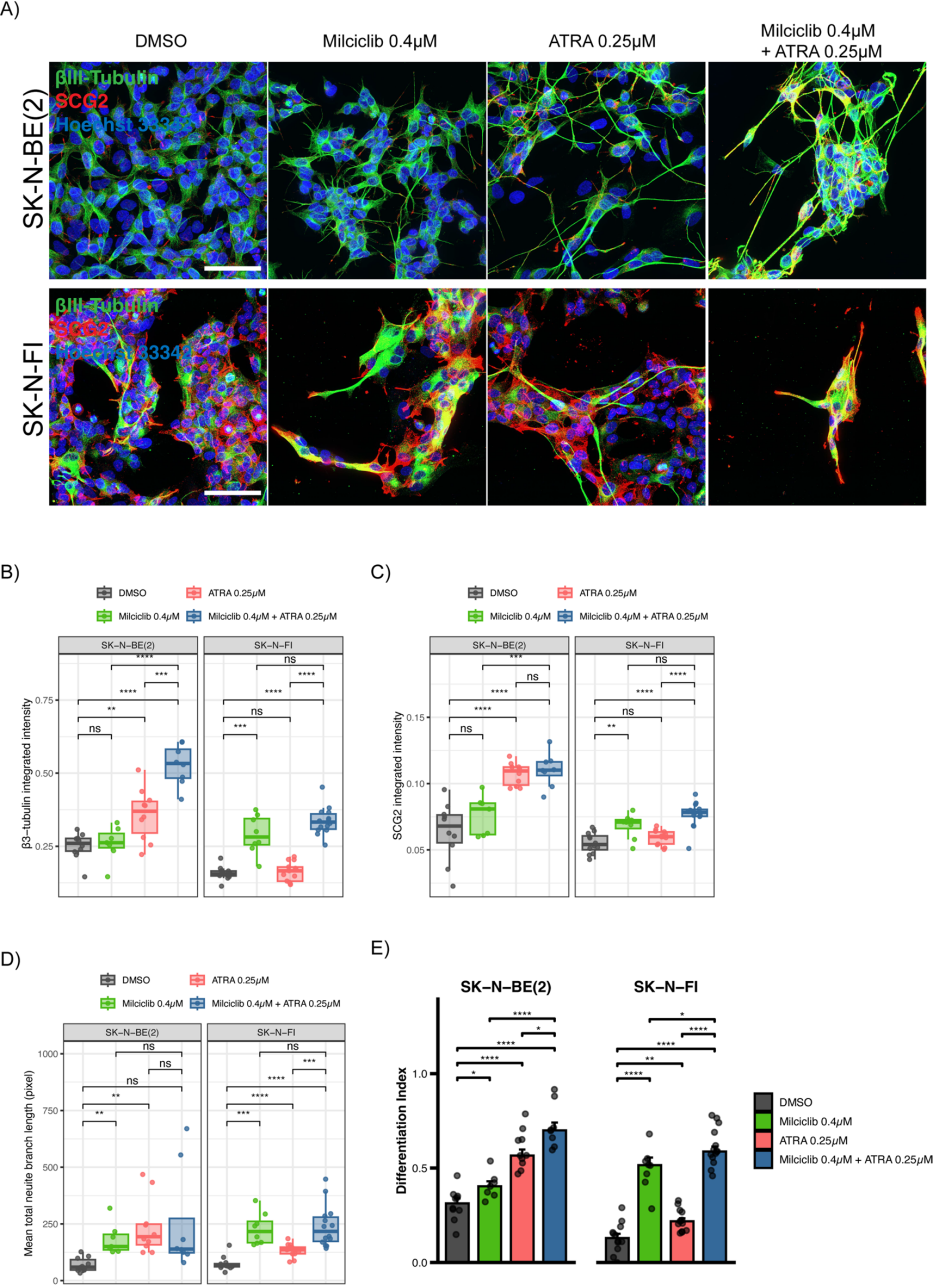
Combination of milciclib and ATRA inhibitors enhances neuronal differentiation of NB cells. (**A**) Immunofluorescent staining of SCG2 (Red), β3-Tub (Green), and DNA (Hoechst 33342) in *MYCN*-amplified SK-N-BE(2) and *MYCN*-non-amplified SK-N-FI cells following 6-days treatment with indicated concentrations of milciclib and ATRA. (**B-C**) Quantification of β3-Tub (**B**) and SCG2 (**C**) integrated signal intensity in Fig. 8A. (**D**) Quantification of mean total neurite length in Fig. 8A. (**E**) Differentiation index of SK-N-BE(2) and SK-N-FI combining SCG2 and β3-Tub intensities, as well as neurite length. DMSO was used as a control treatment. The p-value was considered significant at *p* < 0.05. (*N* ≥ 3, *n* ≥ 6, median ± SEM).

Differentiation therapy using retinoids including ATRA is a standard maintenance treatment for high-risk neuroblastoma patients who have been subjected to intensive chemotherapy/stem cell transplants as means to force remaining cancer cells differentiate into neuronal cells, and to reduce recurrence risk^14^. To this end, we addressed whether CDK2 inhibition could be a potential new approach to enhance differentiation therapy by ATRA in NB. Thus, we evaluated the impact of milciclib and ATRA on SCG2 and β3-Tub intensity, as well as neurite length as described above to define a differentiation index for single and combination treatment using the *MYCN*-amplified SK-N-BE(2) and *MYCN*-non-amplified SK-N-FI cell lines in response to 6-days treatment (Fig. 8A). Treatment with milciclib increased β3-Tub intensity significantly in SK-N-F1 cells, and a trend to increased intensity in SK-N-BE(2) cells (Fig. 8B). Similarly, ATRA treatment resulted in significantly increased β3-Tub intensity in SK-N-BE(2) cells, and a trend to increased intensity in the SK-N-F1 cells (Fig. 8B). Combination of milciclib and ATRA significantly increased β3-Tub intensity in both cell lines, which increase was even more prominent in SK-N-BE(2) cells (Fig. 8B). Milciclib increased SCG2 identity in both cell lines when compared to DMSO, but this increase was higher in SK-N-FI cells, while ATRA treatment increased SCG2 intensity significantly in SK-N-BE(2) cells (Fig. 8C). Combination treatment also significantly increased SCG2 intensity in both cell lines when compared to DMSO control treatment, but this increase remained at comparable levels observed after either single treatment (Fig. 8C). As for neurite length, single treatment significantly increased neurite length when compared to DMSO (Fig. 8D). However, combination treatment did not further increase neurite length when compared to single treatment (Fig. 8D). When compared to DMSO control treatment, all treatment settings significantly increased the differentiation index in SK-N-BE(2) and SK-N-FI cells (Fig. 8E). Notably, combination treatment resulted in a significantly higher differentiation index when compared to single treatment in both cell lines (Fig. 8E). Collectively, these findings suggest that combination of milciclib and ATRA enhances neural differentiation in NB cells that is worth exploring further in the future. It may potentially represent a promising new approach for differentiation therapy during maintenance treatment of high-risk NB patients with *MYCN*-amplification.

## Discussion

Cyclin dependent kinase 2 (CDK2) is a serine/threonine protein kinase and an important regulator of cell cycle progression by controlling the G1/S transition, as well as other biological processes such as DNA repair, chromatin biology and gene transcription, protein stability and degradation, and cellular senescence^62–65^. Previous studies using genetic depletion or pharmacological inhibition of CDK2 suggest that this could be a promising therapeutic target in NB, especially for tumors with *MYCN*-amplification^41–44^. However, how CDK2 contributes to NB tumorigenesis is not fully understood. In the present study, we explored the clinical and biological significance of CDK2 in NB, as well as potential functional crosstalk with MYCN in NB. We show that high CDK2 expression in NB tumors is indicative of high-risk and advanced disease, as well as marker of poor prognosis. We report a previously uncharacterized role of CDK2 as a negative regulator of neuronal differentiation in NB.

Based on our in-silico analysis of gene expression datasets of primary NB tumors, we uncovered that CDK2 expression correlates with advanced stage and progressive NB, high-risk disease, and *MYCN*-amplification. Notably, high CDK2 was indicative of poor survival in NB, and CDK2 expression negatively correlated with high expression of genes related to neuronal differentiation, rendering it a potential marker for the aggressive undifferentiated or poorly differentiated forms of NB. This was further supported by our findings using publicly available single cell RNA-seq data, where CDK2 was expressed in NB sub-populations characterized by cycling cells and *MYCN*-amplification/expression. Although not all the cells even in these clusters overexpressed CDK2, the differentiated NB clusters showed an inverse pattern characterized by lack of CDK2 expression. We further confirmed this by analyzing publicly available gene expression data that had been gathered following CDK2-KD in the *MYCN*-amplified IMR32 cell line^41^, and in our analysis demonstrated enrichment of neuronal differentiation genes among the overexpressed genes. Such correlation between human CDK2 and neuronal differentiation gene expression has not been reported so far to the best of our knowledge in human NB. By pathway analysis, CDK2 expression level was also connected to modulation of genes related to neuronal differentiation, in addition to genes for cell cycle, metabolic pathways, ribosomal biogenesis, and immune pathways. Intriguingly, such a gene expression signature has been suggested to be modulated by MYCN in NB^60,66,67^. Notably, both high MYC/N pathway activity and suppression of neuronal differentiation are linked to aggressive NB phenotype^68^. Collectively, these findings are consistent with our observations suggesting that CDK2, when co-expressed with MYCN, serves as a novel marker of undifferentiated NB.

Functionally, the effects of knockdown or pharmacological inhibition of CDK2 on neuronal differentiation were confirmed by morphological examination of neurite outgrowth, by q-PCR analysis of expression of differentiation marker genes, and by immunofluorescent staining of neuronal differentiation markers. Targeting CDK2 as well as other CDKs has gained great interest in combating cancer in recent years, particularly in MYC/N-driven tumors including NB^43,44,50,69–72^. CDK2 inhibition using different inhibitors has been previously shown to induce cell death in NB cell lines with high MYCN protein levels^41–43,71^. Like these findings, our study also demonstrates that CDK2 depletion using siRNA or inhibition using milciclib or CYC065 induced cell death in *MYCN*-amplified NB cell lines, suggesting that loss of CDK2 activity causes a mix of neuronal differentiation and apoptosis in this subtype of NB. Our findings that CDK2-KD induces apoptosis in *MYCN*-amplified NB agree with Molenaar et al. 2009 demonstrating that CDK2 is synthetic lethal in NB cells with high MYCN expression^41^. The impact of CDK inhibition on neuronal differentiation in NB cell lines has been suggested by a few studies^69,72,73^. Kranenburg et al., 1995 suggested that inhibition of CDK2 or CDK4 induces neuronal differentation in mouse NB cell lines possibly via modualtion of Rb phosphoryaltion^73^. Recently, CDK inhibitors have been demonstrarted as promising differentation agents in humn NB^69,72^. Specifically, taregting CDK4/6 by palbocilcib induces neuronal differentiation in human NB cell lines^69^. This finding was further supported by a study using another CDK4/6 inhibitor Abemaciclib, pan CDK inhibitor Dinaciclib, and CDK2/9 inhibitor Fadraciclib (also known as CYC065 used in our study)^72^. Our study further extends these findings and demonstrates that specific CDK2-KD, and its pharmacological inhibition induces neuronal differentiation both in human *MYCN*- and non-*MYCN*-amplified NB cells.

In this study, we showed that MYC and E2F target genes were down-regulated following CDK2-KD. Reduced expression of E2F target genes in response to CDK2-KD is reminiscent of recent observations reporting induced neuronal differentiaiton in NB cell lines by the CDK4 inhibitor palbociclib^69^. Notably, both CDK4 and CDK2 phosphorylate and inactivate RB^74^, which is an upstream regulator of E2F. MYCN has previously been implicated in regulating NB differentiation^68^, and our results suggest that MYCN pathway downregulation following CDK2-KD or pharmacological CDK2 inhibition could be a potential underlying mechanism impacting neuronal differentiation. Whether the same applies when inhibiting CDK4 is an interesting question that demands further investigation. Phosphorylation of MYC at Ser-62 by ERK stabilizes MYC^58^ and phosphorylation of the same site by CDK2 has been shown to be required for MYC’s ability to bind promoter regions of target genes involved in suppression of RAS-induced cellular senescence and subsequent modulation of their expression in primary fibroblasts^48^. Neuronal differentiation resembles cellular senescence in a sense that cells in both cases exit the cell cycle and stop proliferating while maintaining metabolic activity. This raised the question whether CDK2 depletion/inhibition also affects MYCN Ser-54 phosphorylation (the equivalent of MYC Ser-62) and MYCN protein stability in NB. Our data on this was ambiguous. CDK2-KD reduced MYCN Ser-54 phosphorylation but not total MYCN levels at 48 h of treatment in IMR32, it reduced both phosphorylation and MYCN total levels in SK-N-BE(2) cells, while the pharmacological CDK2 inhibitors reduced MYCN levels in most NB cell lines with MYCN-amplification including IMR32. One should bear in mind that regulation of MYC protein phosphorylation and stability around Ser-62 is a complex process, where Ser-62 phosphorylation (and induced stabilization) is very transient and rapidly results in phosphorylation of Thr-58 and subsequent FBXW7-mediated degradation, depending on the status of FBXW7, the GSK3β kinase (phosphorylating Thr-58) and phosphatases and de-ubiquitination enzymes acting at these sites^75^. Future studies are required to resolve how this regulation works for MYCN in NB.

In this study we show that MYCN depletion reduces *CDK2* mRNA and protein levels and based on MYCN ChIP-Seq data analysis we uncover that the *CDK2* gene promoter is occupied by MYCN overlapping with four MYC E-boxes, suggesting that the *CDK2* gene is a direct MYCN target gene. These findings point to a positive feedback loop between MYCN and CDK2 in NB, suggesting the existence of an oncogenic MYCN/CDK2 axis with potential therapeutic value. Notably, treatment with the BET-bromodomain inhibitor JQ1^59^ or the MYC/N: MAX inhibitor MYCMI-7^29,60^ recapitulated the impact of CDK2 inhibition on the induction of neuronal differentiation in the *MYCN*-amplified cell lines. We also demonstrate that combinations of CDK2 and MYC/N inhibitors result in increased anti-proliferative effects against NB cell lines in vitro. In this study, combinations of milciclib and JQ1 induced synergetic or additive effects irrespective of *MYCN*-amplification status, while combination of milciclib and MYCMI-7 induced additive effects. We also show a trend that combinations of milciclib and MYCMI-7 further enhanced neuronal differentiation in NB cell lines over single inhibitor treatment, although this did not reach statistical significance. This suggests that CDK2-MYCN axis enhances NB cell growth, in part, by suppressing neuronal differentiation. Further, combination treatments lead to stronger reduction in CDK2 and MYCN levels when compared to single treatments. These observations are in line with previous observations demonstrating combination of CDK2 and BET inhibitors as promising anti-tumor strategy in MYC-driven medulloblastoma models^50^. Thus, these observations argue in favor of combinatorial CDK2 and MYC inhibitor treatment to effectively inhibit the MYC/N pathway and combat MYC/N driven tumors. In addition, we found that CDK2 inhibition enhanced ATRA-induced neuronal differentiation, suggesting that inhibition of CDK2 could potentially be a new component in the RA-based differentiation therapy that is currently used as maintenance treatment in NB.

In conclusion, this study shows that depletion or pharmacological inhibition of CDK2 induces neuronal differentiation both in *MYCN*- and non-*MYCN*-amplified NB cells. Further, we demonstrate that MYCN binds the CDK2 promoter and regulates CDK2 transcription Moreover, it suggests that CDK2-inhibitors combined with MYC inhibitors or ATRA is a potential new therapeutic strategy in NB.

### Limitations of the study

The four human NB cell lines used in this study were chosen to represent NB based on the *MYCN*-amplification status. It would be interesting to expand the impact of CDK2 inhibition alone or in combination with MYC/N inhibitors and ATRA on a larger panel of NB cell lines representing more genetic backgrounds in the future to strengthen our findings. Another limitation of the study is that the CDK2 inhibitors used are not highly specific for CDK2 - milciclib is a CDK2/TrkA inhibitor and CYC065 is a CDK2/9 inhibitor. This is a general problem in the CDK inhibitor field, and although the results were confirmed by CDK2 knockdown in NB cell lines, hopefully more selective CDK inhibitors will be developed in the future. Finally, to provide clinical ‘’ translational’’ impact of this study, future studies in mouse models of neuroblastoma are needed to evaluate the differentiation therapy potential of CDK2 inhibitors in an in vivo setting.

## Materials and methods

### In-silico analysis of publicly available gene expression data of primary NB samples and cell lines, single cell-RNA-seq, and MYCN ChIP-seq

The R2: Genomics Analysis and Visualization Platform was used to investigate the clinical and prognostic value of CDK2 expression in four different NB patient cohort studies; the SEQC-498, Westermann-579, Kocak-649, and TARGET-161 datasets. All figures were generated using the R2 platform built-in functions. Background correction and normalization of expression values of the *MYCN*-amplified cell line IMR32 gene expression array (GSE16480)^41^ was analyzed using AFFY package in R package^76^. Differential expression between experimental conditions was ascertained using linear models implemented in the *limma*package. Genes with adjusted p-value < 0.05 and fold change greater than 1 were considered significantly differentially expressed. *CDK2*, *MYCN*, and *MYC* expression state and dynamics in single cell RNA-seq of primary NB samples was analyzed using the online tool available at NB atlas (https://single-cell.be/nbatlas/)^52^. Gene ontology and pathway analysis were performed using Gene Set Enrichment Analysis (Broad institute, GSEA version 4.2.2) and Enrichr: a comprehensive gene set enrichment analysis web server^77,78^, and Kyoto Encyclopedia of Genes and Genomes (KEGG) with permission^51^.The R2 genome browser was utilized to show MYCN (ChIP-Seq) enrichment at the transcription start site of *CDK2* gene.

### Cell culture

A set of human authenticated STR typed and mycoplasma free neuroblastoma cell lines representing *MYCN*-amplified and *MYCN*-non-amplified status was used in this study. *MYCN*-amplified cell lines IMR32, SK-N-BE(2), and SK-N-DZ, as well as TET21N cell line with doxycycline-regulatable *MYCN* expression were grown in RPMI-1640 medium (Gibco), while *MYCN*-non-amplified cell line SK-N-FI and SH-SY5Y were grown in DMEM medium (Gibco) supplemented with 10% fetal bovine serum (FBS, Gibco) and 1% penicillin streptomycin (Gibco) in a humidified incubator containing 5% CO_2_ at 37 °C. Human NB cell line cultures were checked for mycoplasma negativity regularly (once a month) using MycoAlert^®^PLUS Mycoplasma Detection Kit (Lonza, Catalog #: LT07-710).

### Chemical inhibitors and compounds used in this study

Milciclib (CDK2/TRKA inhibitor) was purchased from Divbio Science Europe (Catalog #: PHA-848125) and CYC065 (CDK2/9 inhibitor) was kindly provided by Cyclacel Pharmaceuticals (New Jersey, USA). The BET-bromodomain inhibitor JQ1 was purchased from Tocris Bioscience (Catalog #: 4499) and the MYC: MAX inhibitor MYCMI-7 was kindly provided by MyCural Therapeutics (Uppsala, Sweden). All-*trans*-retinoic acid (ATRA) was purchased from Sigma-Aldrich (Catalog #: R2625). All compounds were dissolved in DMSO at 10 mM stock concentration. Doxycycline hyclate from Sigma-Aldrich (Catalog#: D9891) was dissolved in water at 10 mg/ml stock concentration. Puromycin solution of 10 mg/ml stock concentration was purchased from Invivogen (Catalog #: ant-pr-1).

### Western blot

Western blots were performed as described previously^60^. Primary antibodies used for immunoblotting were anti-MYCN (SC53993, Santa Cruz Biotechnology, RRID: AB_831602), anti-c-MYC (ab56, Abcam, RRID: AB_304976), anti-CDK2 (ab32147, Abcam, RRID: AB_726775), anti-pS54-MYCN (A300-206 A, Bethyl, RRID: AB_2235736), anti-Actin (A5441, Sigma, RRID: AB_476744), anti-cleaved-PARP (9546, Cell Signaling, RRID: AB_2160593), anti-phospho Rb (retinoblastoma protein) (phospho T821, ab4787, Abcam, RRID: AB_304624), anti-Rb (sc-50, Santa Cruz, RRID: AB_632339). Secondary antibodies were Anti-rabbit HRP-conjugated (ab97080, Abcam, RRID: AB_10679808) and anti-mouse HRP-conjugated (ab97046, Abcam, RRID: AB_10680920). Quantification of all western blots was performed using ImageJ software. Actin band was used for normalization in all the of the quantification.

### Resazurin (cell viability) assay

Four thousand cells were seeded in a 96-well plate overnight, then treated with increasing concentration of Milciclib and CYC065, JQ1 or MYCMI-7 for 72 h. For combination treatments, cells were with single or different combinations of CDK2 and MYC inhibitors at indicated concentrations for 72 h. Treatment with DMSO was used as control. Cell viability was measured by adding resazurin solution to evaluate metabolic activity, incubated for 2–3 h at 37 °C, then resazurin conversion of resorufin was measured at absorbance at 570 nm. Cell viability reads were used for further analysis to measure the efficacy of the drug combinations using SynergyFinder web-application for interactive analysis and visualization of multi drug, multi-dose combination response data^61^. Synergy score of the different drug combinations was calculated using the Bliss synergy models. Synergy score of less than − 10 indicates antagonism; from − 10 to 10 indicates additive effects; while synergy score larger than 10 suggests synergistic action.

### SiRNA transfection

Neuroblastoma cell lines were transfected using lipofectamine 2000 (Life Technologies, Catalog#: 11668027) with non-targeting siRNA (Life Technologies, Catalog#: 462001), siRNA against CDK2 (Life Technologies, Catalog#: VHS40359) or MYCN (Life Technologies, Catalog#: HSS106841) for 3 days following manufacturer’s protocol. In brief, cells were seeded in 6-well plates O/N. At 50% confluency, cells were transfected with 100 pmol of each siRNA in serum free media overnight, then medium was refreshed.

### Generation of stable inducible CDK2 knock-down NB cell lines

IMR32, SK-N-BE(2), SK-N-FI, and SH-SY5Y cell lines were transduced with TRIPZ Inducible Lentiviral vectors expressing red fluorescent protein (RFP)-tagged shRNA against human CDK2 shRNA; shCDK2-I04 (Catalog #: RHS4696-200699438) or shCDK2-I08 (Catalog #: RHS4696-201904769), or a TRIPZ Inducible FRP-tagged non-targeting control (Catalog ID: RHS4743) from Dharmacon, GE Life Sciences. Stably transduced cell lines were under puromycin selection for 10 days and were further assessed by the expression of RFP following doxycycline treatment (1 µg/ml) for 3 and 6 days.

### Neuronal differentiation, image analysis and qRT-PCR

The impact of CDK2-KD or pharmacological inhibition on the differentiation of neuroblastoma cell lines was evaluated by microscopic examination of neurite extension and by qPCR analysis of a set of neuronal differentiation markers identified in this study at 6 days post-knockdown or 72 h post-treatment with milciclib or CYC065. Treatment with ATRA was used as control treatment of neuronal differentiation. JQ1 and MYCMI-7 treatments were also performed as proxy for MYCN/MYC inhibition. RNA extraction, cDNA synthesis, and qPCR were performed as described before^60^. Primers for qPCR used in this study are provided in supplementary table S2. Cell morphology was observed using an AxioObserver microscope (Zeiss) equipped with a Colibri7 illumination system (Zeiss) and a Hamamatsu C11440 camera (Hamamatsu). Image acquisition was performed in the czi format and subsequently converted to 8-bit.tiff format using ZenBlue software. Number of neurite extensions in brightfield images was manually counted in ImageJ software.

### Immunofluorescence staining, image analysis, and quantitative analysis

NB cell lines were cultured in 96-well plates containing polymer coverslips (89601 IbiTreat; Ibidi, Germany), treated as indicated in each figure for 6-days. Cells were fixed with 4% paraformaldehyde for 10 min at room temperature (RT), then were permeabilized with 0.2% Triton X-100 in PBS for 10 min at RT, washed with PBS, and blocked for 1 h in blocking buffer consisting of 0.1% Triton X-100 and 5% goat serum (5425 S; Cell Signaling Technology) in PBS. For immunostaining, cells were incubated overnight at 4 °C with rabbit anti-SCG2 (1:400, HPA011893; Atlas Antibodies, RRID: AB_1856656) and mouse anti-βIII-tubulin (1:50–1:200, ab7751; Abcam, RRID: AB_306045) antibodies diluted in blocking buffer. After washing with PBS, Alexa Fluor 647–conjugated goat anti-rabbit and Alexa Fluor 488–conjugated goat anti-mouse secondary antibodies (1:500, Invitrogen), together with Hoechst 33,342 (1:1000, H1399; Invitrogen), were applied for 2 h at RT. Following multiple PBS washes, wells were maintained in PBS for imaging. Images were acquired using a Nikon Crest v3 spinning disk confocal microscope controlled by NIS-Elements software. Representative images were collected from at least six replicate images per condition. All.nd2 files were converted to 16-bit.tiff format, with each channel exported as an individual image using NIS-Elements software. Cell segmentation was performed using Cellpose^79^ and feature extraction was conducted using CellProfiler version 4.2.1^80^. To quantify differentiation-related properties, the MeasureObjectSizeShape, MeasureObjectSkeleton, and MeasureObjectIntensity modules were employed. For each well and experimental condition, per-cell features were aggregated to compute a composite differentiation index based on neurite morphology and marker intensity (SCG2 and β3-Tub). Selected features were z-score standardized and averaged, and the resulting differentiation index was subsequently min–max normalized to a 0–1 scale.

### Statistical analysis

For in-silico analysis of gene expression data of NB cohort studies analyzed in this study, statistical analysis was performed based on R2 platform built-in one-way ANOVA. Bonferroni correction of raw *p*-values was used for the Kaplan–Meier-analysis of overall and event free survival curves. Two-tailed, unpaired Student *t*-test was used to calculate the statistical significance of cell viability data, qRT-PCR, and quantification of the number of neurite outgrowths in single treatment in all bright field images. One-way ANOVA with multiple comparisons in GraphPad was used to evaluate the statistical significance of the number of neurite outgrowth in Fig. 7. For immunofluorescence-based quantitative analyses, image-derived measurements were analyzed in RStudio using the tidyverse and ggplot2 packages. Data were analyzed separately for each cell line and treatment condition. Pre-specified pairwise two-sample Student t-tests were used to assess differences between treatment groups, with no adjustment applied for multiple comparisons. Data are presented as median ± SEM or as boxplots, where each point represents measurements derived from a single image.

## Supplementary Information

Below is the link to the electronic supplementary material.

Supplementary Material 1

Supplementary Material 2

Supplementary Material 3

## Data Availability

The NB primary tumor gene expression datasets: the SEQC-498, Westermann-579, Kocak-649, and TARGET-161 analyzed during the current study are available in the R2: Genomics Analysis and Visualization Platform repository ([http://r2.amc.nl](http:/r2.amc.nl)). The gene expression dataset following CDK2-KD in the MYCN-amplified cell line IMR32 is available at gene expression omnibus under the accession number GSE16480. The single cell RNA-seq data is available at NB atlas (https://single-cell.be/nbatlas/). No datasets were generated in this study.
