# Supplementary material for: CDK2 inhibition promotes neuronal differentiation in neuroblastoma
Source: Sci Rep. 2026 Feb 6;16:5255. doi: 10.1038/s41598-026-38123-4 (PMC12881557; doi:10.1038/s41598-026-38123-4)

Supplementary Table S2: sequences of qPCR primers used in this study.

Primer	Sequence 5'-3'
GAP43-For	GAGCAGCCAAGCTGAAGAGAAC
GAP43-Rev	GCCATTTCTTAGAGTTCAGGCATG
MEGF8-For	CGGAAAATGGCTTCAACCAGCAG
MEGF8-Rev	CTCGTGGTAGACAGCAGAGTGA
SLIT2-For	CAGAGCTTCAGCAACATGACCC
SLIT2-Rev	GAAAGCACCTTCAGGCACAACAG
STMN2-For	CCAGAAGAAACTGGAGGCTGCA
STMN2-Rev	GCTTTTCCTCCGCCATCTTGCT
SHANK3-For	AGGATCACACCCGCCGAGATTA
SHANK3-Rev	CTACAGACTTGGTCCGTGGAATC
CDK2-For	ATGGATGCCTCTGCTCTCACTG
CDK2-Rev	CCCGATGAGAATGGCAGAAAGC
MYCN-For	ACCACAAGGCCCTCAGTACCTC
MYCN-Rev	TGACAGCCTTGGTGTTGGAGGA
GAPDH-For	TCGGAGTCAACGGATTTGGT
GAPDH-Rev	TGAAGGGGTCATTGATGGCA

Supplementary Figures

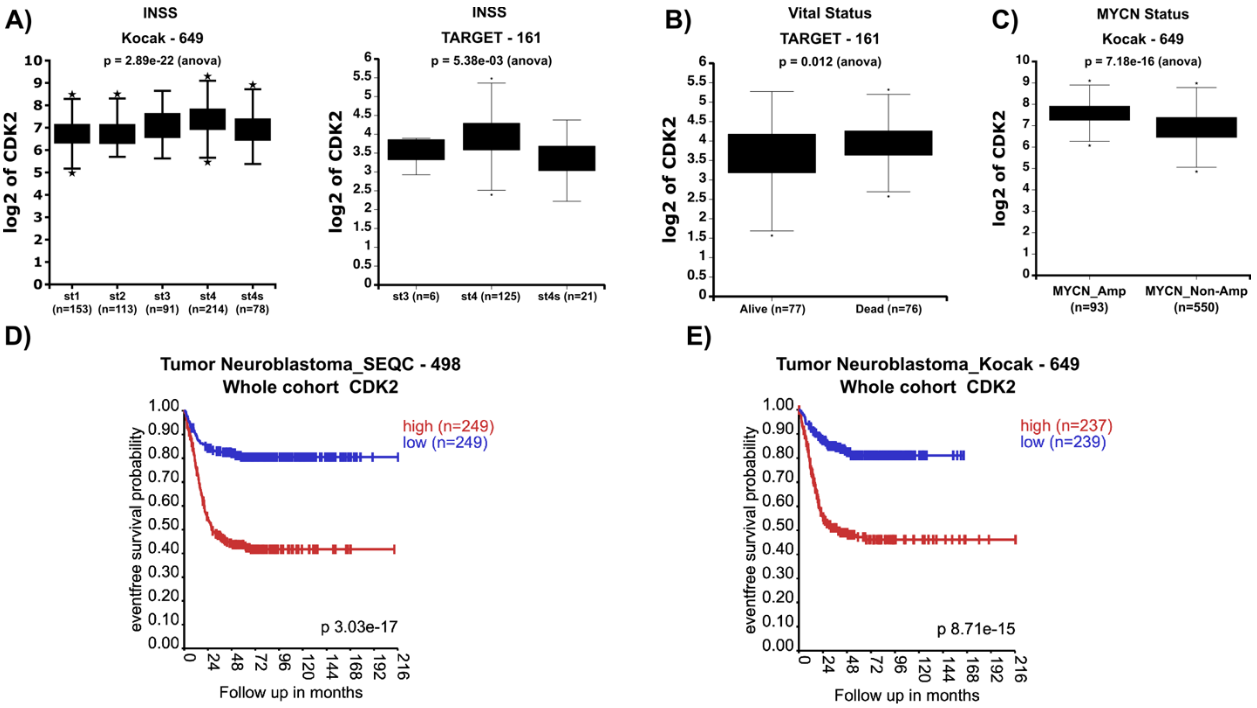

Supplementary Figure 1. High CDK2 expression levels correlate with high-risk neuroblastoma, MYCN-amplification, and poor prognosis. A) Log2 of CDK2 mRNA levels in different NB stages as defined by The International Neuroblastoma Staging System (INSS) in the Kocak-649 and TARGET-161 cohort studies. B) Log2 of CDK2 mRNA levels in NB tumors of alive and dead patients in the TARGET-161 cohort. C) Log2 of CDK2 mRNA levels in the *MYCN*-amplified vs. *MYCN*-non-amplified NB patient subgroups in the Kocak-649 cohort. (D-E) Kaplan–Meier-analysis of event-free survival datasets based on CDK2 expression of NB patients in the SEQC-498 (D) and KOCAK-649 (E). Statistical significance was calculated using R2-Genomics built-in one-way ANOVA for correlation of CDK2 mRNA levels with clinical parameters, and Bonferroni correction of raw *p*-values for survival analysis.

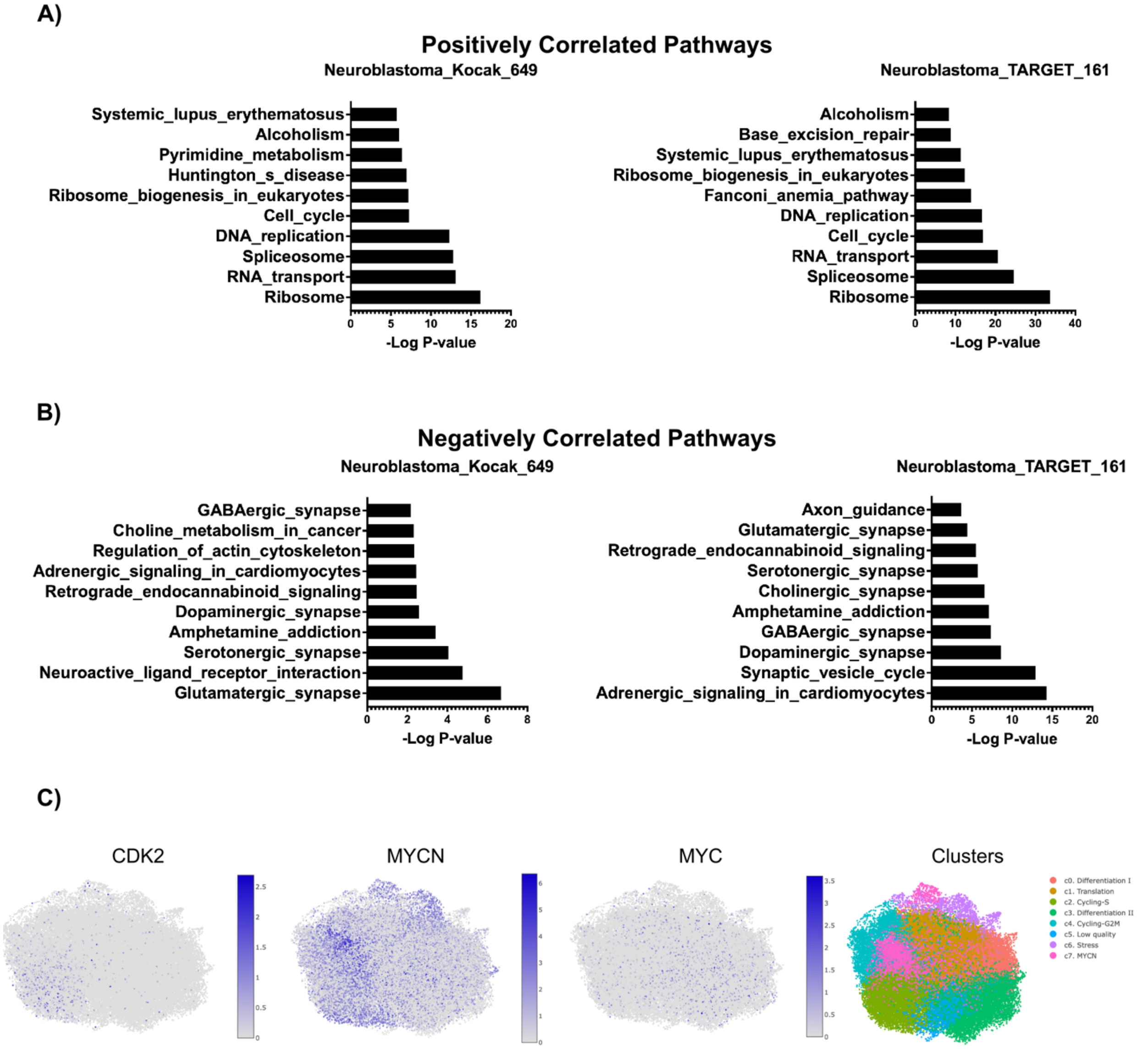

Supplementary Figure 2. CDK2 expression correlates with undifferentiated phenotype in NB. A-B) Kyoto Encyclopedia of Genes and Genomes (KEGG) of genes whose expression positively (A) and negatively (B) correlated with CDK2 in the KocakK-649 and TARGET-161 NB cohort studies. Statistical significance was calculated using R2-Genomics built-in one-way ANOVA . C) Analysis of CDK2, MYCN, and MYC expression level in single cell RNA-seq data available at NBAtlas: A harmonized single-cell transcriptomic reference atlas of human neuroblastoma (https://single-

cell.be/nbatlas/) in the NB tumor subset. The last panel represent NB tumor cell clusters as defined by gene expression signature.

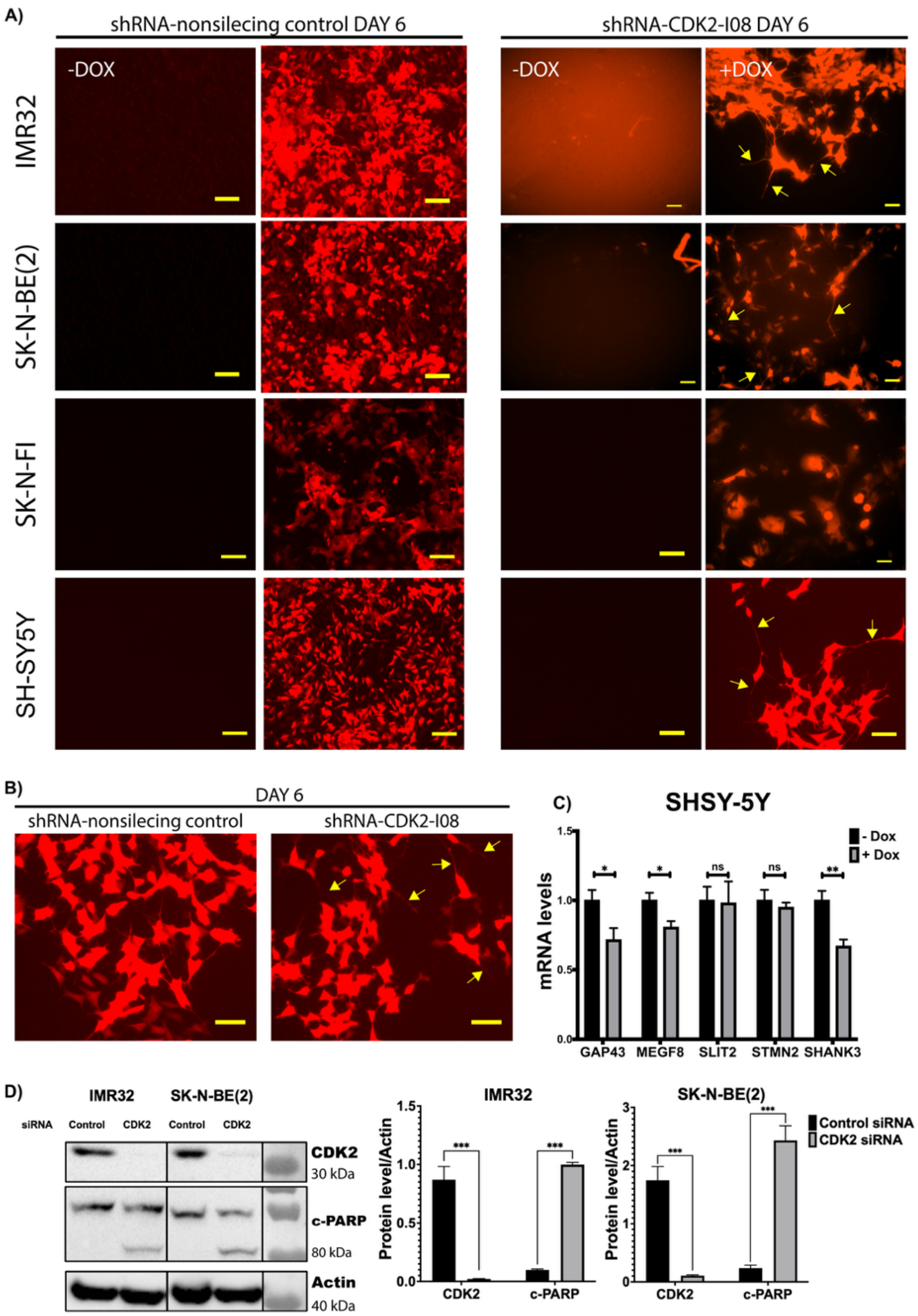

Supplementary Figure 3. CDK2-KD induces neuronal differentiation in NB cell lines. A) monitoring RFP signal and neurite outgrowth in response to 6-day treatment with 1 mg/ml doxycycline in IMR32, SK-N-BE(2), SK-N-FI, and SH-SY5Y cell lines expressing shRNA-control or shRNA-CDK2-I08. B) RFP and cell morphology of SH-SY5Y cell line expressing shRNA-control or shRNA-CDK2-I08 following 6-day treatment with 1 mg/ml doxycycline. Scale

bars represent 50 μm. Arrow heads indicate neurite outgrowth. C) qRT-qPCR analysis of neuronal differentiation markers in shRNA-CDK2-I08 expressing SH-SY5Y cell line treated with 1 mg/ml doxycycline for 6 days. GAPDH was used as a housekeeping gene for qRT-qPCR. Mock H_2O treatment was used as control. D) Western blot analysis and quantification of CDK2 and cleaved-PARP in IMR32 and SK-N-BE(2) cell lines transfected with control-or CDK2-siRNA for 72 hours. Error bars represent the standard deviation of three independent biological experiments. p-value was calculated using two-tailed, unpaired Student t-test in GraphPad prism. * p-value ≤ 0.05; ** p-value ≤ 0.01; *** p-value ≤ 0.001. Actin was used as loading control for western blots.

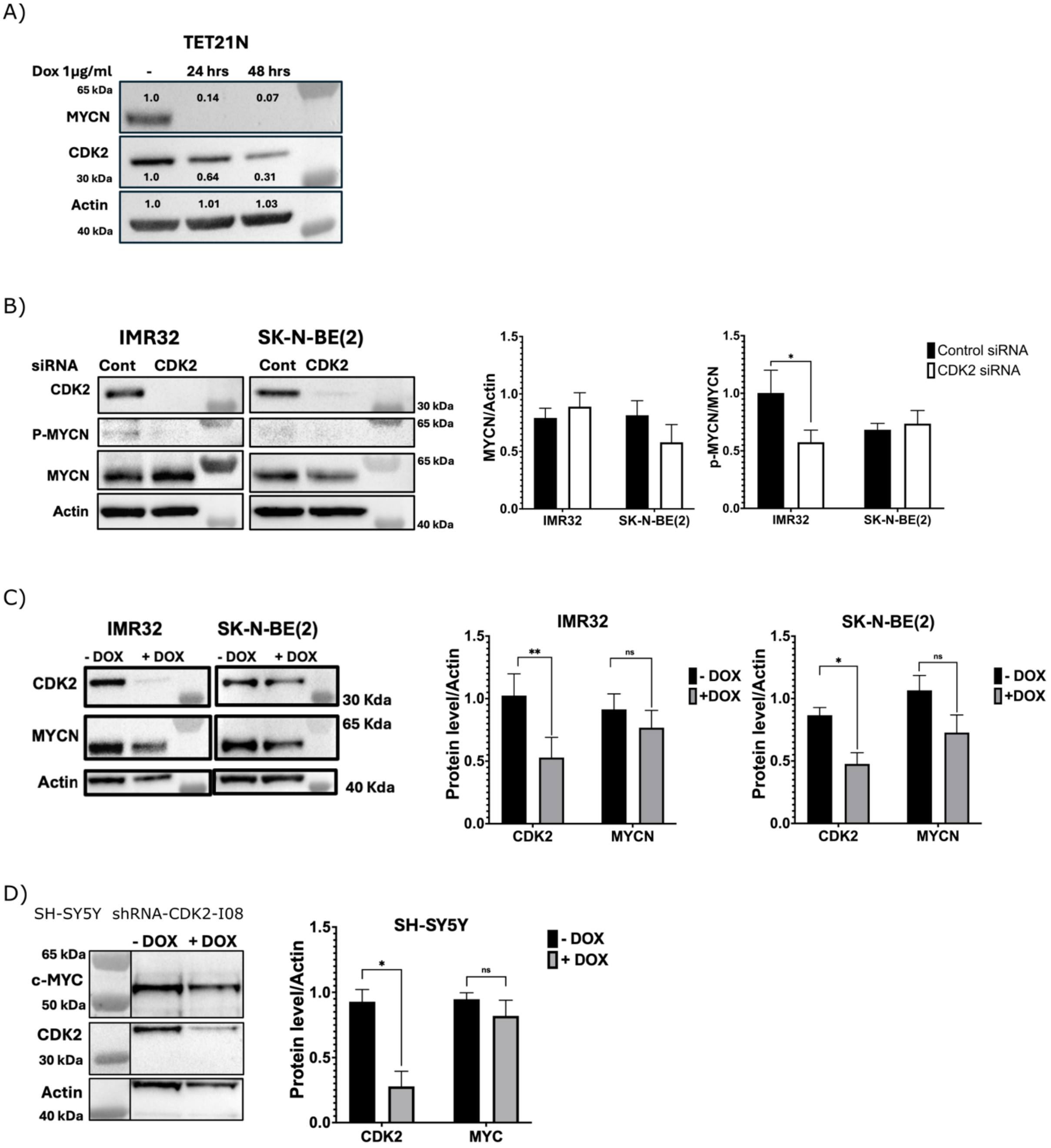

Supplementary Figure 4. CDK2 is a MYCN target gene in neuroblastoma and modulates the MYC pathway. A) Westen blot analysis and quantification of CDK2 and MYCN protein levels in TET21N cell line in response to 24 and 48 hrs 1 mg/ml doxycycline treatment. B) Analysis of CDK2, MYCN, MYCN Ser54-phosphorylation levels in IMR32 and SK-N-BE(2) cell lines following 48 hrs transfection with control- or CDK2-siRNA. The bars measure total MYCN/Actin

levels and the ratio of MYCN phosphorylation at Ser54/MYCN. Error bars represent the standard deviation of at least three independent biological experiments. C) Westen blot analysis and quantification of CDK2 and MYCN protein levels in the *MYCN*-amplified cell lines IMR32 and SK-N-BE(2) stably transduced with CDK2 shRNA-I08 treated with H_2O or 1 mg/ml doxycycline for 3 days. D) Westen blot analysis and quantification of CDK2 and MYCN protein levels in the *MYCN*-non-amplified cell line SH-SY5Y stably transduced with CDK2 shRNA-I08 treated with H_2O or 1 mg/ml doxycycline for 3 days. Error bars represent the standard deviation of three independent biological experiments. p-value was calculated using two-tailed, unpaired Student t-test in GraphPad prism. * p-value ≤ 0.05; ** p-value ≤ 0.01; *** p-value ≤ 0.001. Actin was used as loading control for western blots.

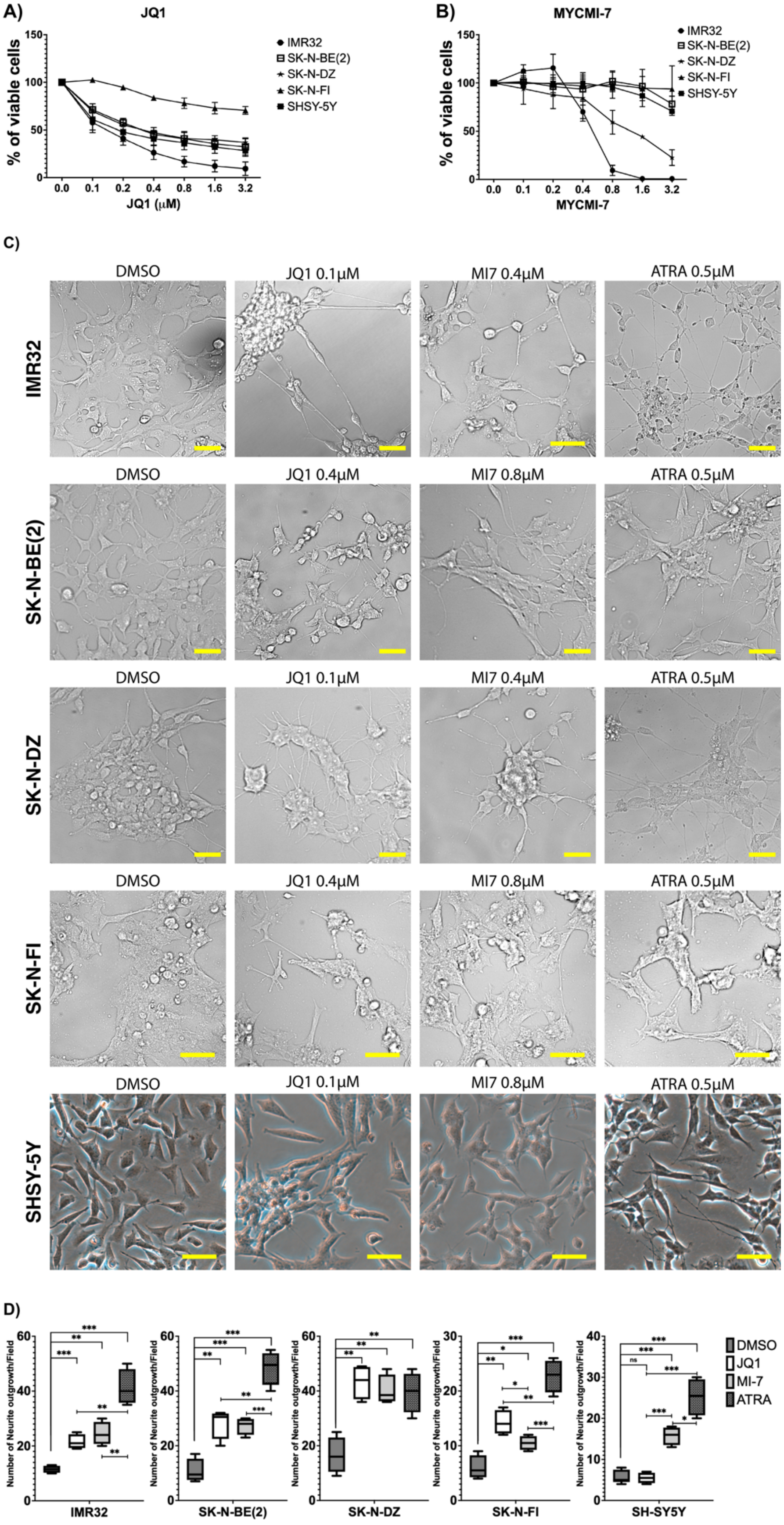
A)
JQ1
% of viable cells
JQ1 (μM)
IMR32
SK-N-BE(2)
SK-N-DZ
SK-N-FI
SHSY-5Y
B)
MYCMI-7
% of viable cells
MYCMI-7
C)
DMSO
JQ1 0.1μM
MI7 0.4μM
ATRA 0.5μM
IMR32
JQ1 0.4μM
MI7 0.8μM
SK-N-BE(2)
SK-N-DZ
SK-N-FI
SHSY-5Y
D)
Number of Neurite outgrowth/Field
DMSO
JQ1
MI-7
ATRA
IMR32
SK-N-BE(2)
SK-N-DZ
SK-N-FI
SH-SY5Y

Supplementary Figure 5. MYC/N inhibition induces neuronal differentiation in NB cell lines. A-B) Cell viability analysis of human NB cell lines used in this study in response to 72 hours treatment with range of JQ1 (A) and MYCMI-7 (B) concentrations. DMSO was used as control treatment. Error bars represent the standard deviation of three independent biological experiments. C) Bright field images of IMR32, SK-N-BE(2), SK-N-DZ, SK-N-FI and SH-SY5Y cell lines following 6-day treatment with DMSO and indicated concentrations of JQ1, MYCMI-7, and ATRA. Please note that DMSO and ATRA images are the same used in Figure 4D since all treatments and analyses belonged to the same experiment. Scale bars represent 50 µm. D) Quantification of neurite outgrowth in all cell lines as indicated in Figure S4D, which was manually counted using ImageJ software. Bars represent the median number of neurite extensions per field of three independent biological experiments. The p-value was calculated using two-tailed, unpaired Student t-test in GraphPad Prism. * p-value $\leq$ 0.05; ** p-value $\leq$ 0.01; *** p-value $\leq$ 0.001.

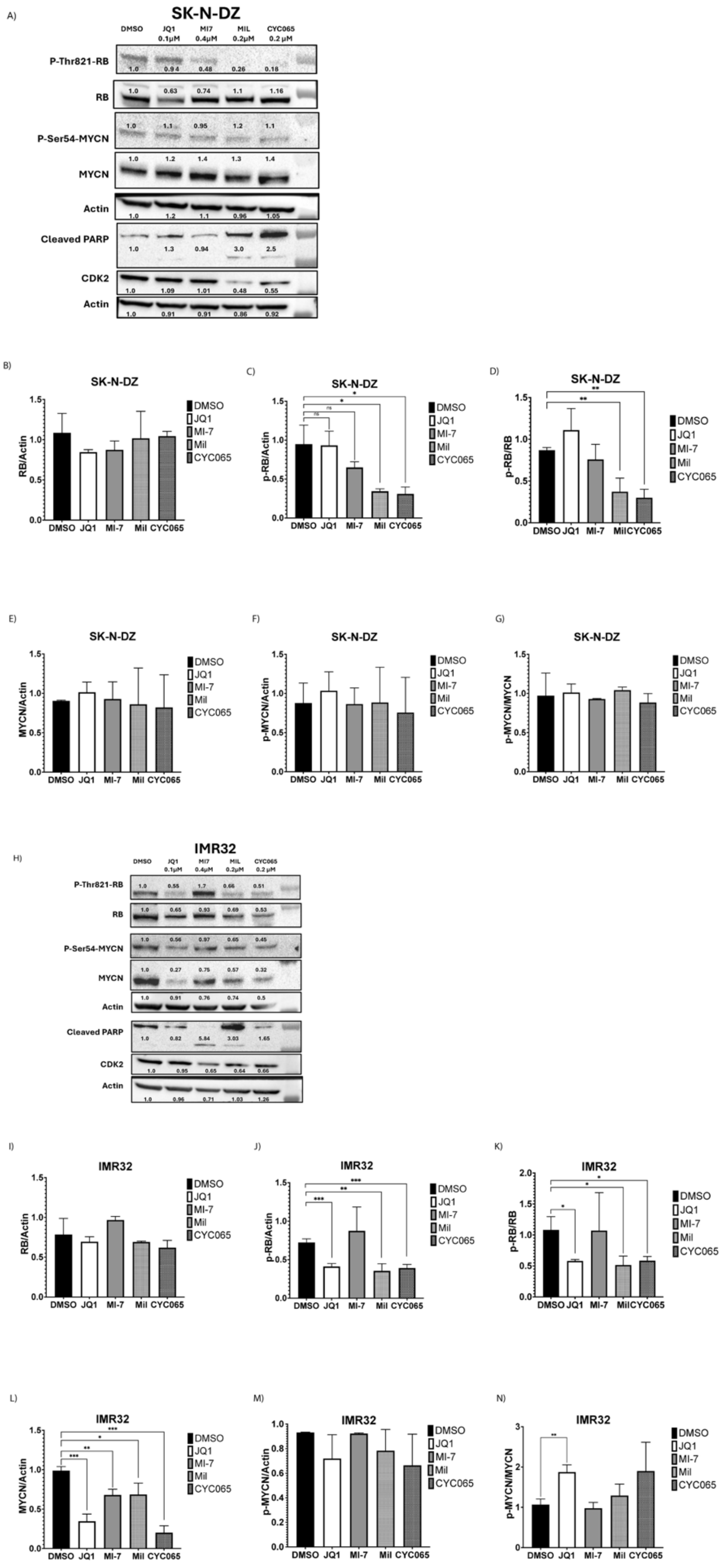
A)
SK-N-DZ
DMSO
JQ1 0.1μM
MI7 0.4μM
MIL 0.2μM
CYC065 0.2 μM
P-Thr821-RB
RB
P-Ser54-MYCN
MYCN
Actin
Cleaved PARP
CDK2
Actin
B)
SK-N-DZ
RB/Actin
C)
SK-N-DZ
p-RB/Actin
D)
SK-N-DZ
p-RB/RB
E)
SK-N-DZ
MYCN/Actin
F)
SK-N-DZ
p-MYCN/Actin
G)
SK-N-DZ
p-MYCN/MYCN
DMSO
JQ1
MI-7
Mil
CYC065
H)
IMR32
DMSO
JQ1 0.1μM
MI7 0.4μM
MIL 0.2μM
CYC065 0.2 μM
P-Thr821-RB
RB
P-Ser54-MYCN
MYCN
Actin
Cleaved PARP
CDK2
Actin
I)
IMR32
RB/Actin
J)
IMR32
p-RB/Actin
K)
IMR32
p-RB/RB
L)
IMR32
MYCN/Actin
M)
IMR32
p-MYCN/Actin
N)
IMR32
p-MYCN/MYCN

Supplementary Figure 6. Western blot analysis and quantification of phosphorylated-Thr821-RB, total RB, phosphorylated-Ser54-MYCN, total MYCN, cleaved PARP, and CDK2 protein levels in SK-N-DZ (A) and IMR32 (H) cell lines following 72 hours treatment with indicated concentrations of JQ1, MYCMI-7, Milciclib, and CYC065. Actin was used as loading control. B-D) Quantification of RB, p-Thr821-RB, and p-RB/RB ration in SK-N-DZ. E-G) Quantification of MYCN, p-Ser45-MYCN, and p-MYCN/MYCN ration in SK-N-DZ. I-K) Quantification of RB, p-Thr821-RB, and p-RB/RB ration in IMR32. L-N) Quantification of MYCN, p-Ser45-MYCN, and p-MYCN/MYCN ration in IMR32. Error bars represent the standard deviation of three independent biological experiments. p-value was calculated using two-tailed, unpaired Student t-test in GraphPad prism. * p-value ≤ 0.05; ** p-value ≤ 0.01; *** p-value ≤ 0.001.

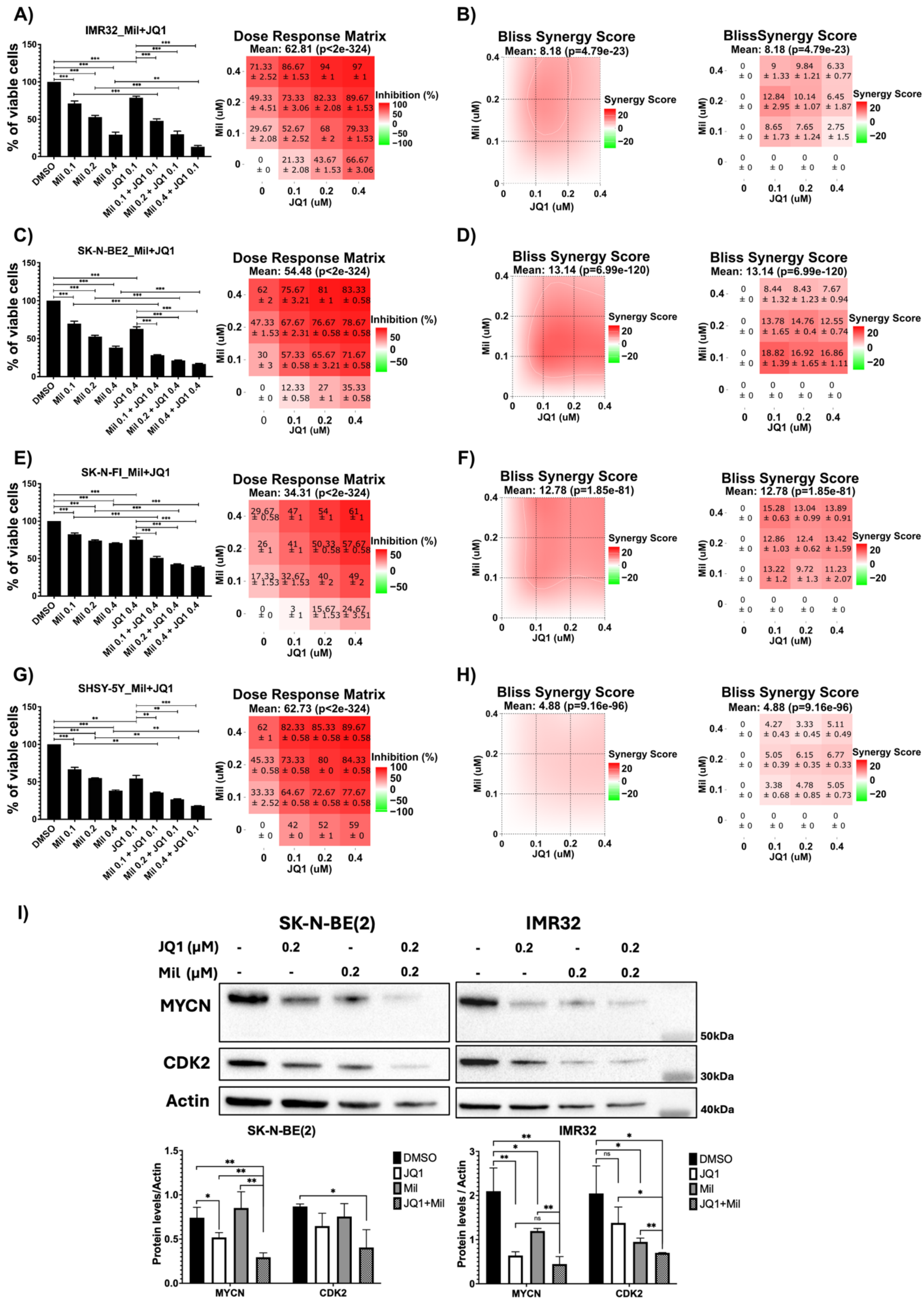

A)
IMR32_Mil+JQ1
% of viable cells
Dose Response Matrix
Mean: 62.81 (p<2e-324)
Inhibition (%)
Mil (uM)
JQ1 (uM)
B)
Bliss Synergy Score
Mean: 8.18 (p=4.79e-23)
Synergy Score
BlissSynergy Score
C)
SK-N-BE2_Mil+JQ1
Dose Response Matrix
Mean: 54.48 (p<2e-324)
D)
Bliss Synergy Score
Mean: 13.14 (p=6.99e-120)
E)
SK-N-FI_Mil+JQ1
Dose Response Matrix
Mean: 34.31 (p<2e-324)
F)
Bliss Synergy Score
Mean: 12.78 (p=1.85e-81)
G)
SHSY-5Y_Mil+JQ1
Dose Response Matrix
Mean: 62.73 (p<2e-324)
H)
Bliss Synergy Score
Mean: 4.88 (p=9.16e-96)
I)
SK-N-BE(2)
IMR32
JQ1 (μM)
Mil (μM)
MYCN
CDK2
Actin
50kDa
30kDa
40kDa
Protein levels/Actin
Protein levels / Actin
DMSO
JQ1
Mil
JQ1+Mil

Supplementary Figure 7. Combinations of Milciclib and JQ1 demonstrate potent inhibitory effects on NB cell lines. A-H) analysis of cell viability, dose-response, and drug synergy score of Milciclib and JQ1 combinations in IMR32 (A-B), SK-N-BE(2) (C-D), SK-N-FI (E-F), SH-SY5Y (G-H) cell lines. (I) Western blot analysis and quantification of MYCN and CDK2 protein levels in SK-N-BE(2) and IMR32 cell lines treated for 72 hrs with indicated concentration of Milciclib and JQ1 in single and combination treatment. Actin was used as loading control. Error bars represent the standard deviation of three independent biological experiments. The p-value was calculated using two-tailed, unpaired Student t-test in GraphPad Prism. * p-value ≤ 0.05; ** p-value ≤ 0.01; *** p-value ≤ 0.001. Synergy score of the different drug combinations was calculated using the Bliss synergy models. Synergy score of less than -10 indicates antagonism; from -10 to 10 indicates additive effects; while synergy score larger than 10 suggests synergistic action.

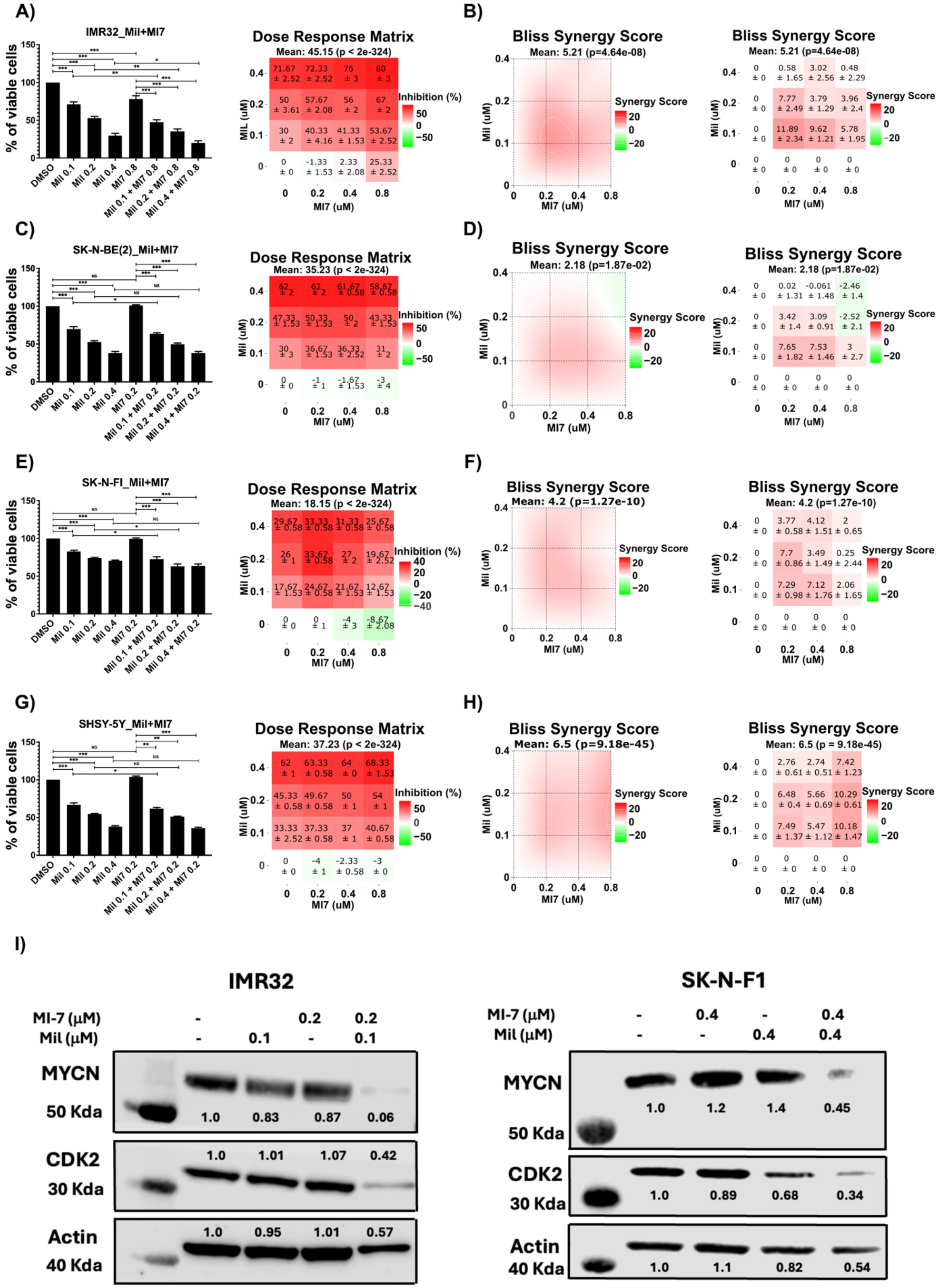

A)
IMR32_Mil+MI7
% of viable cells
Dose Response Matrix
Mean: 45.15 (p < 2e-324)
Inhibition (%)
MI7 (uM)
B)
Bliss Synergy Score
Mean: 5.21 (p=4.64e-08)
Synergy Score
Mil (uM)
C)
SK-N-BE(2)_Mil+MI7
Dose Response Matrix
Mean: 35.23 (p < 2e-324)
D)
Bliss Synergy Score
Mean: 2.18 (p=1.87e-02)
E)
SK-N-FI_Mil+MI7
Dose Response Matrix
Mean: 18.15 (p < 2e-324)
F)
Bliss Synergy Score
Mean: 4.2 (p=1.27e-10)
G)
SHSY-5Y_Mil+MI7
Dose Response Matrix
Mean: 37.23 (p < 2e-324)
H)
Bliss Synergy Score
Mean: 6.5 (p=9.18e-45)
I)
IMR32
MI-7 (µM)
Mil (µM)
MYCN
50 Kda
1.0 0.83 0.87 0.06
CDK2
30 Kda
1.0 1.01 1.07 0.42
Actin
40 Kda
1.0 0.95 1.01 0.57
SK-N-F1
MYCN
1.0 1.2 1.4 0.45
CDK2
1.0 0.89 0.68 0.34
Actin
1.0 1.1 0.82 0.54

Supplementary Figure 8. Combinations of Milciclib and MYCMI-7 demonstrate additive effects in NB cell lines irrespective of *MYCN*-amplification status. Analysis of cell viability, dose response matrix, bliss synergy scores of NB cell lines IMR32 (A-B), SK-N-BE(2) (C-D), SK-N-FI (E-F), and SHSY-5Y (G-H) treated for 72 hours with indicated concentrations of Milciclib and MYCMI-7. I) Western blot analysis and quantification of MYCN and CDK2 protein levels in IMR32 and SK-N-F1 cell lines following 72 hrs treatment with indicated concentrations of Milciclib and MYCMI-7 in single and combination settings. Actin was used as loading control. Error bars represent the standard deviation of three independent biological experiments. The p-value was calculated using two-tailed, unpaired Student t-test in GraphPad Prism. * p-value ≤ 0.05; ** p-value ≤ 0.01; *** p-value ≤ 0.001. Synergy score of the different drug combinations was calculated using the Bliss synergy models. Synergy score of less than -10 indicates antagonism; from -10 to 10 indicates additive effects; while synergy score larger than 10 suggests synergistic action.

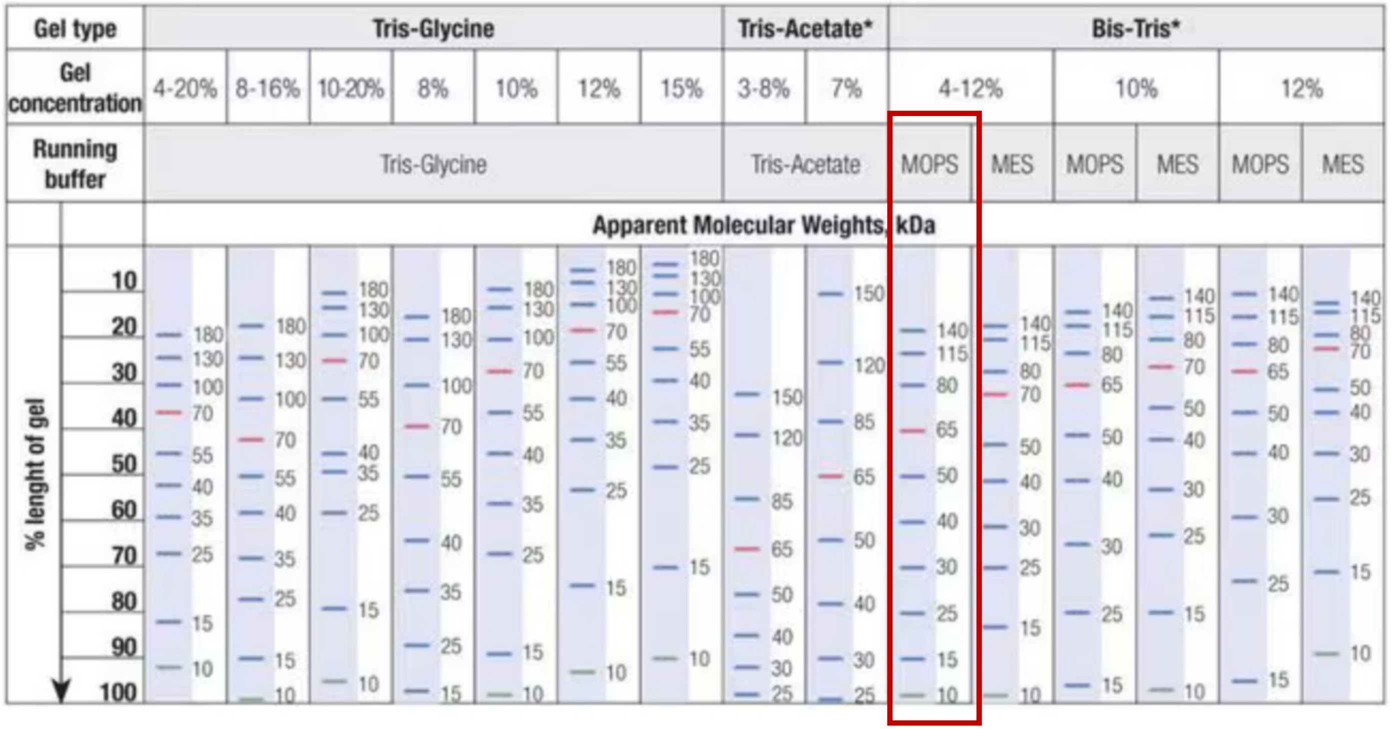

- All westerns were ran using 4-12% Bis-Tris gels in MOPS buffer. PageRuler™ Prestained Protein Ladder , 10 to 180 kDa (Thermo Fisher Catalog#: 26616) was used as Marker. The PageRuler migration pattern is highlighted in red.

- Please note that all membranes were cut around the molecular weight of the analyzed proteins before blocking and incubation with primary antibodies.

- Western blots are arranged according to their listing/appearance order in the manuscript.

Uncropped western blots related to Figure 3A

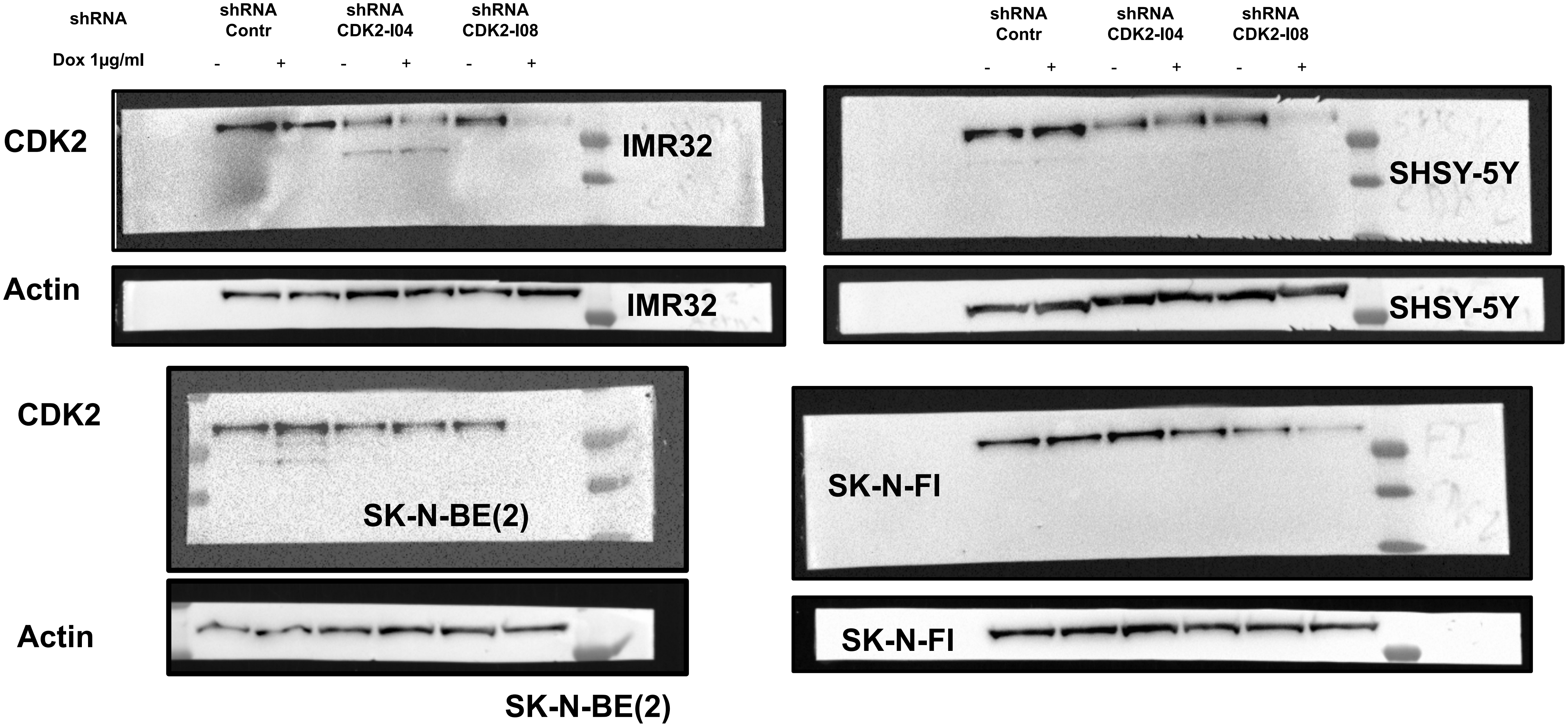

Uncropped western blots related to Figure S3D

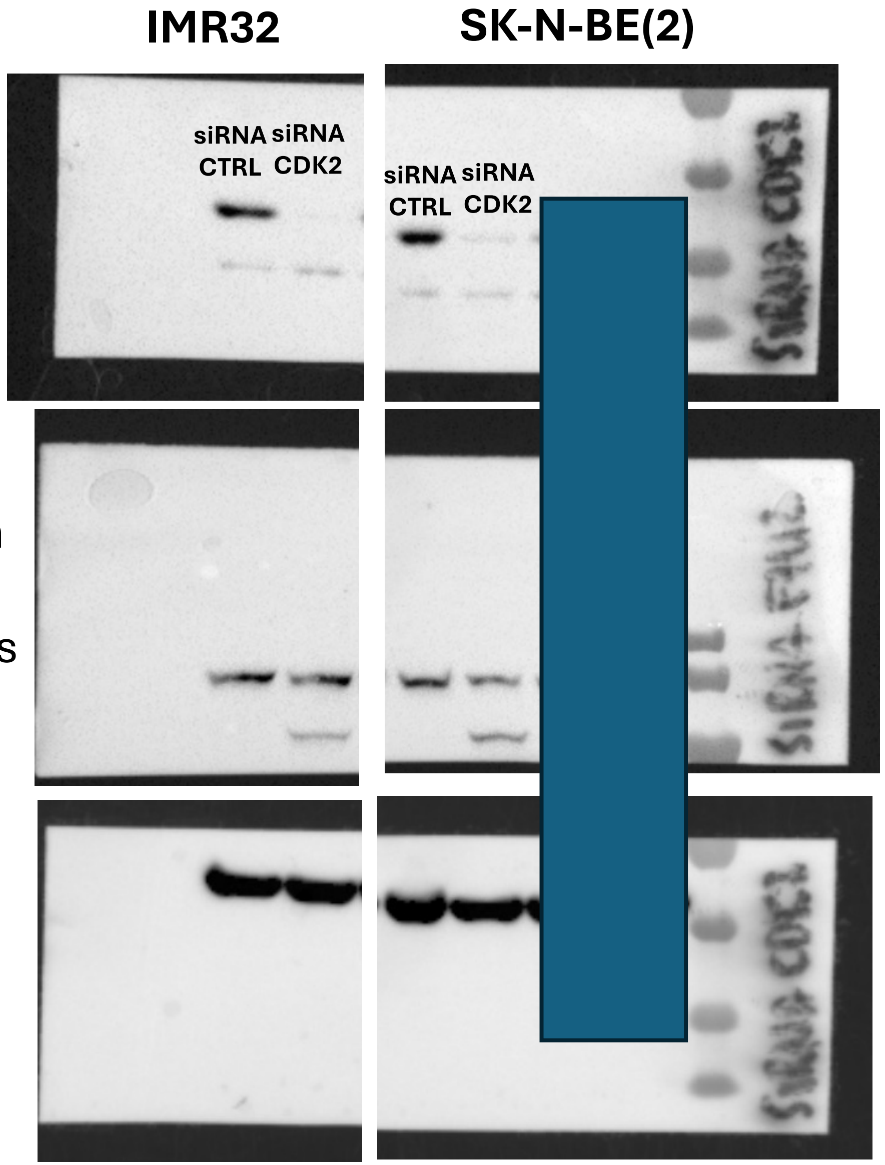

Same membrane for all blots, but in between irrelevant lanes are cropped out

Uncropped western blots related to Figure 4A

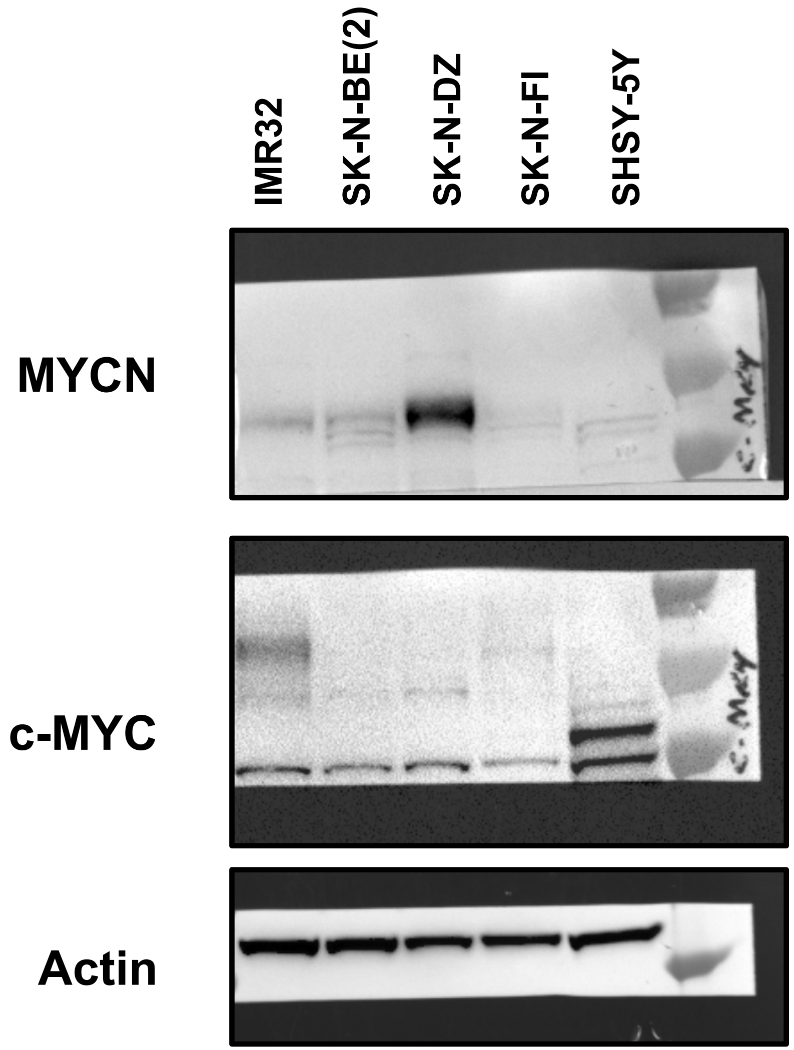

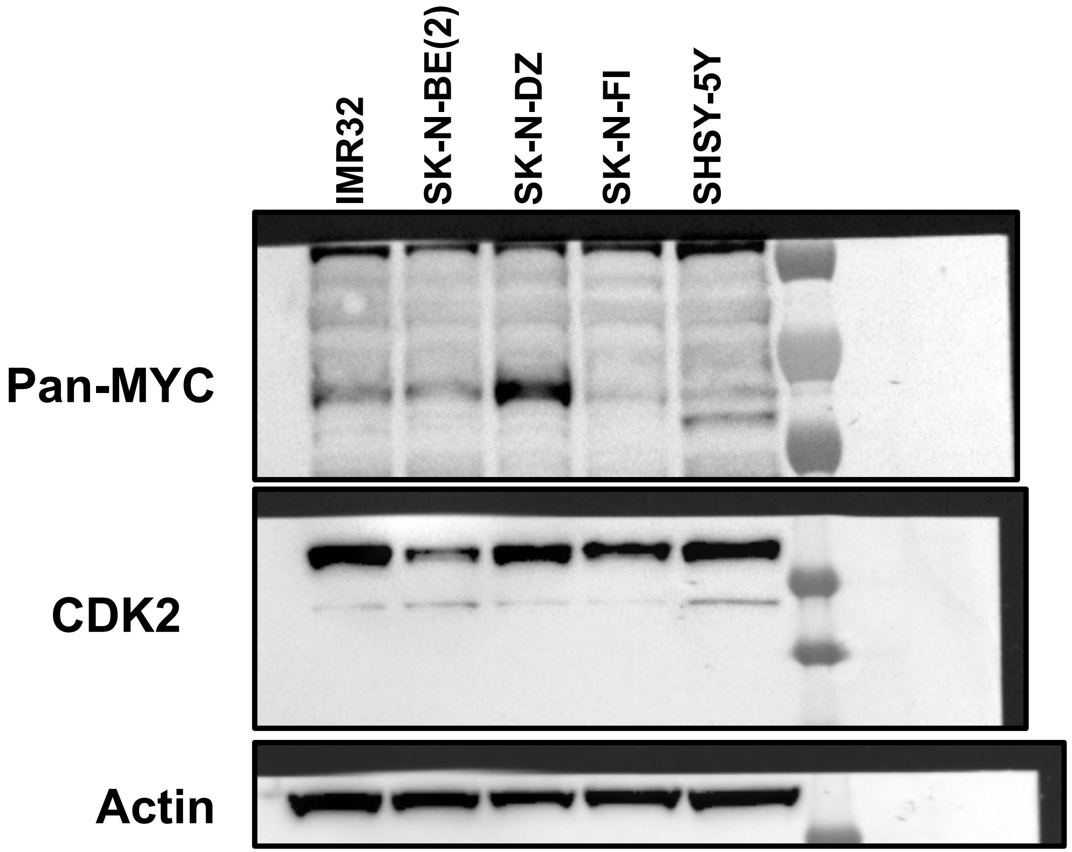

Uncropped western blots related to Figure 6C

IMR32

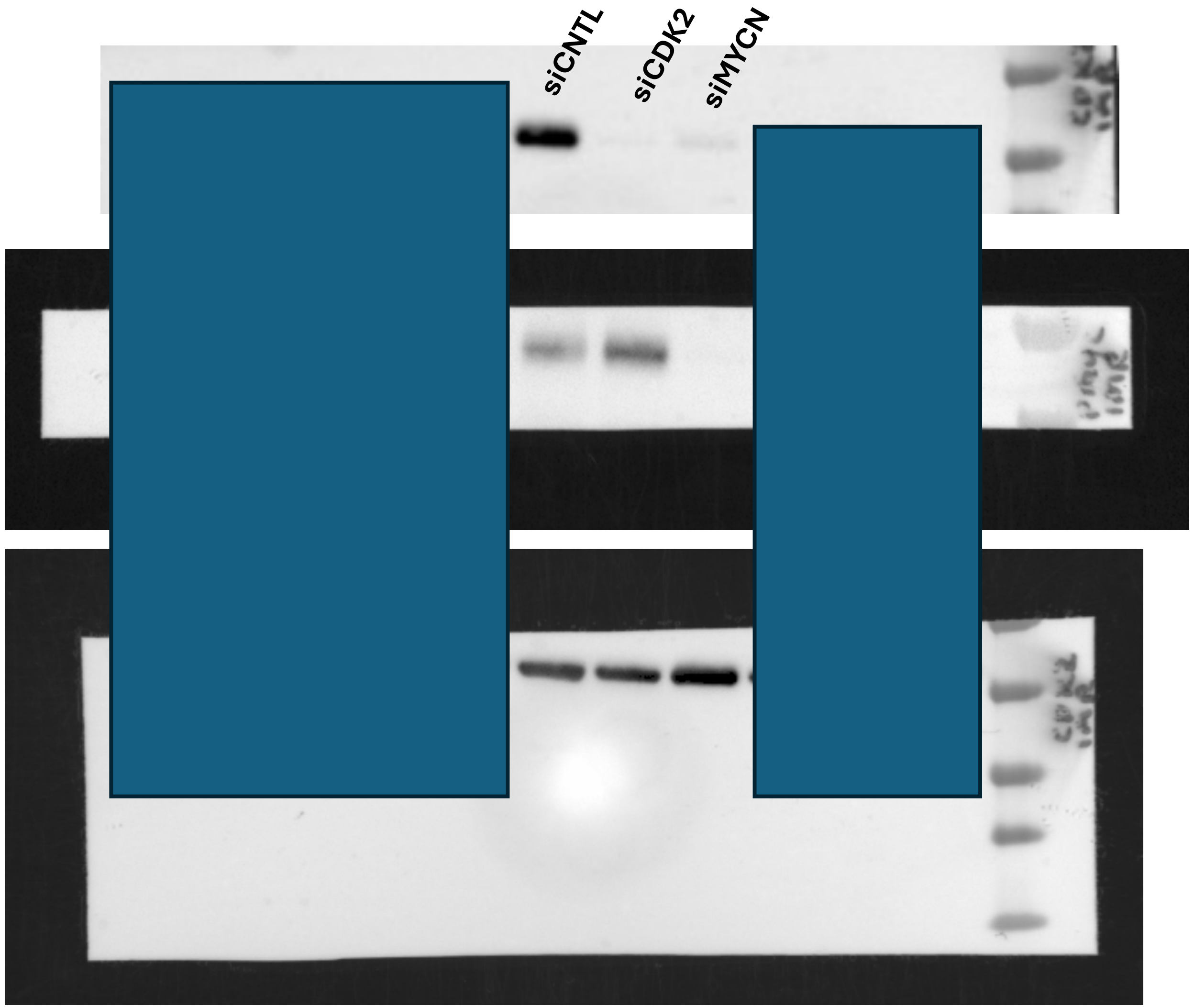

CDK2

MYCN

ACTIN

SK-N-BE2

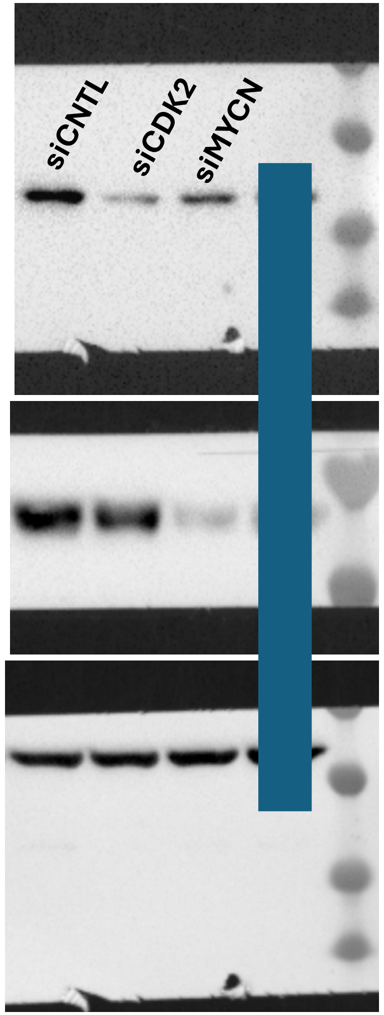

Same membrane for all blots, but in between irrelevant lanes are cropped out

Uncropped western blots related to Figure 6E

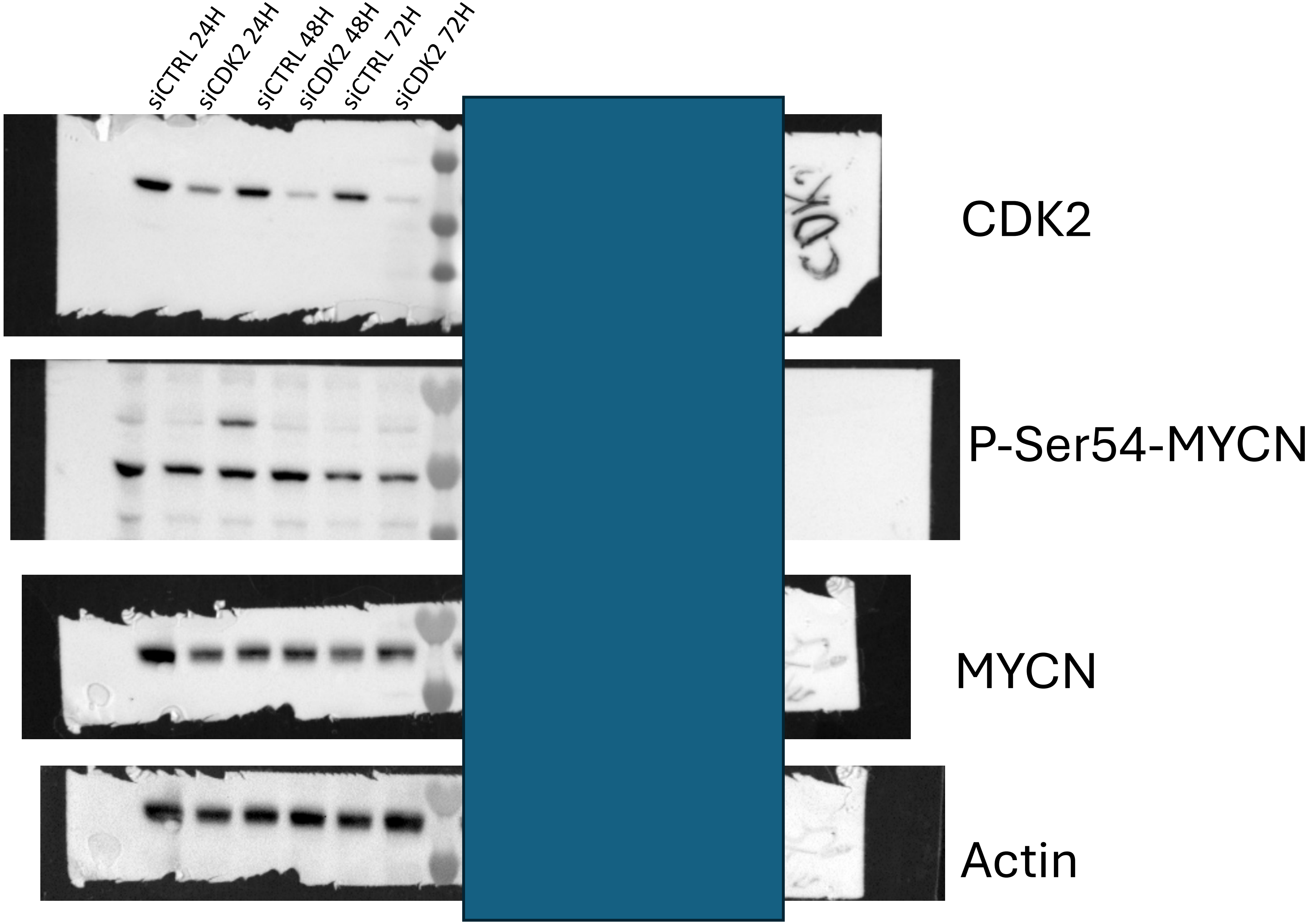

Uncropped western blots related to Figure 4SA

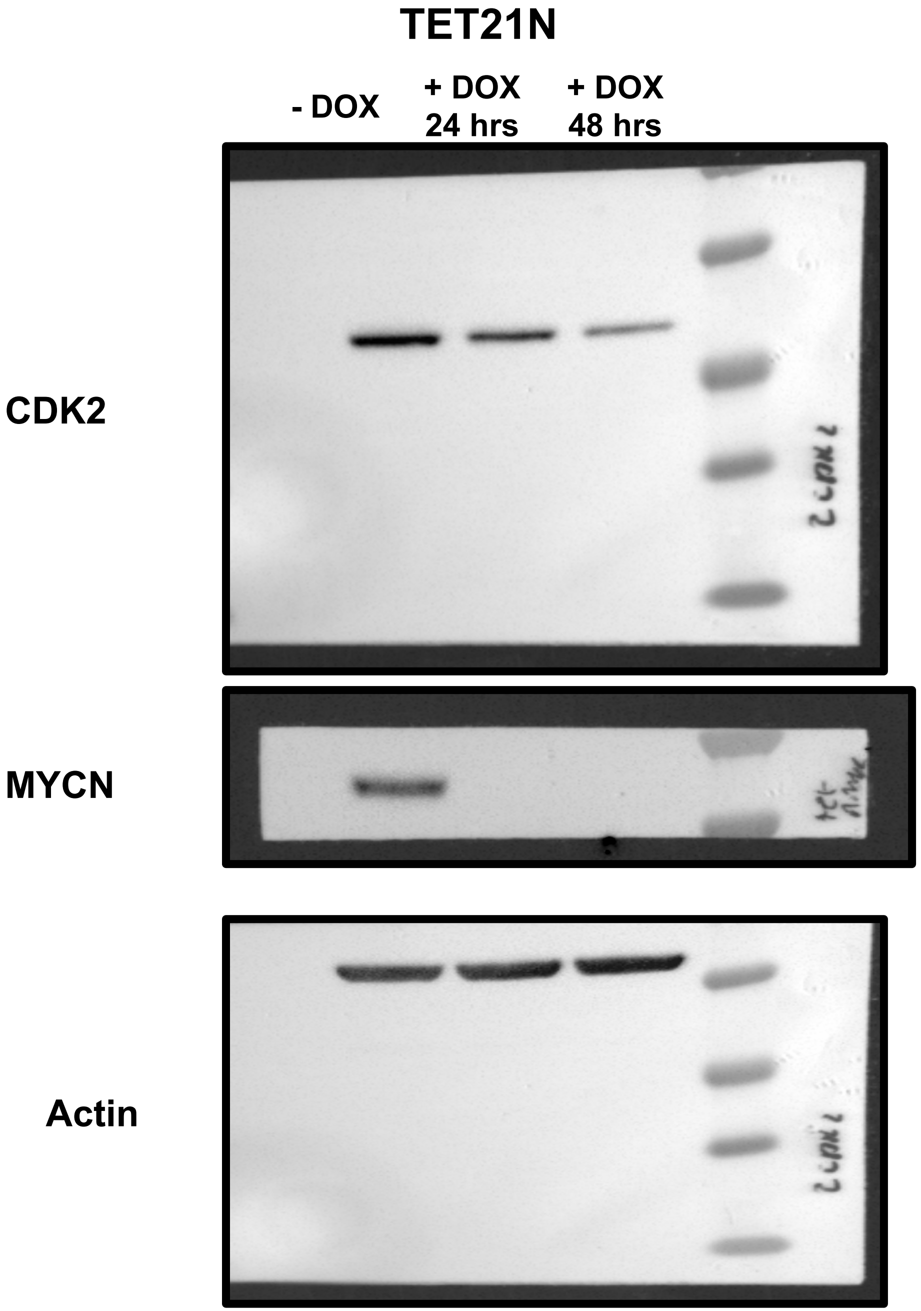

Uncropped western blots related to Figure 4SB

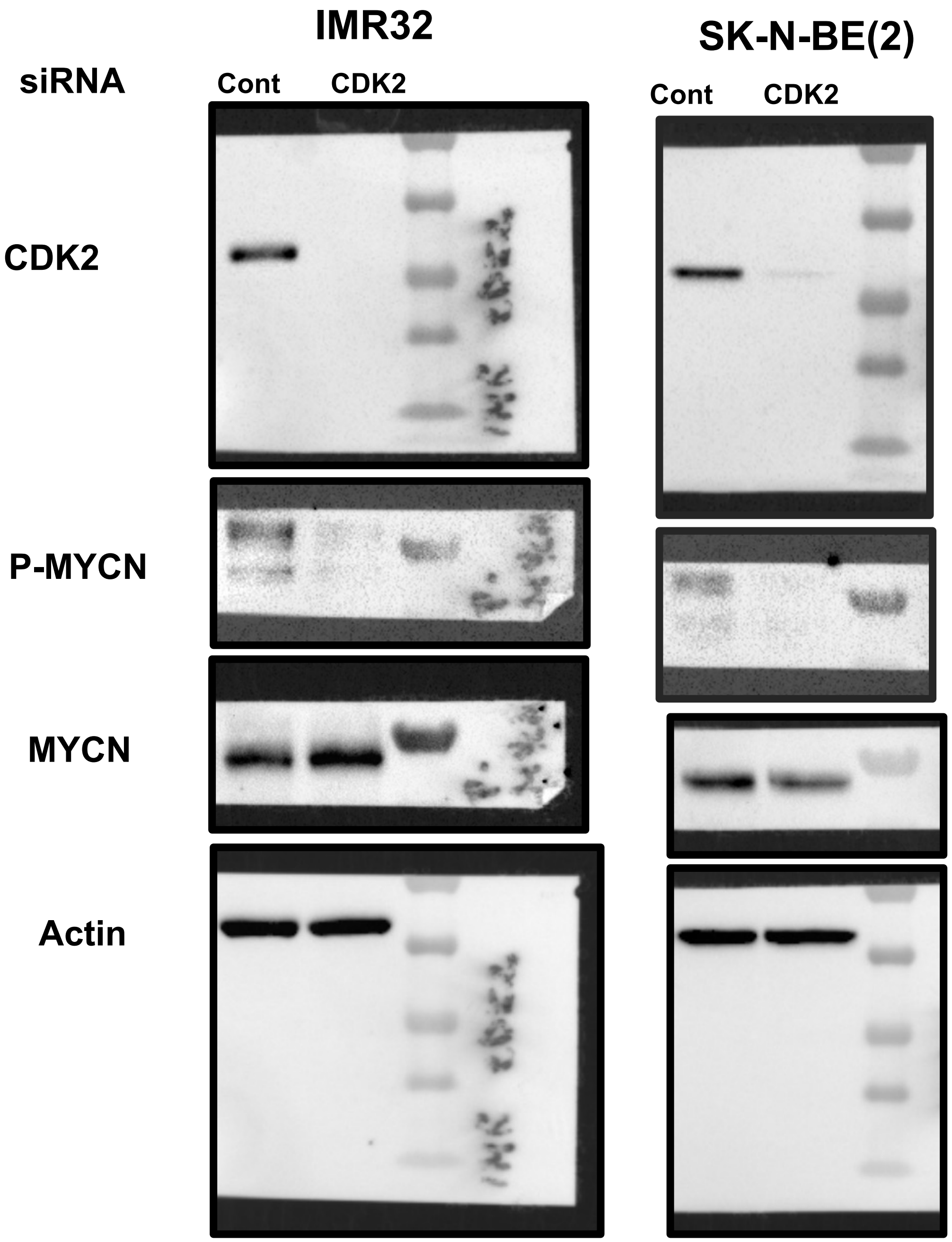

Uncropped western blots related to Figure 4SC

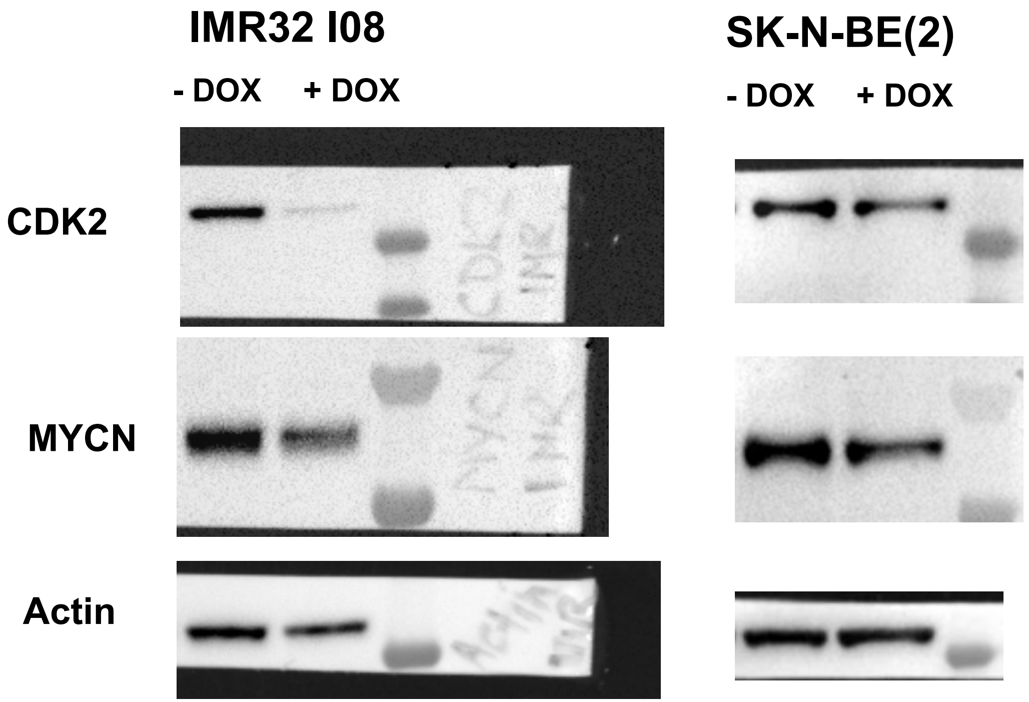

Uncropped western blots related to Figure 4SD

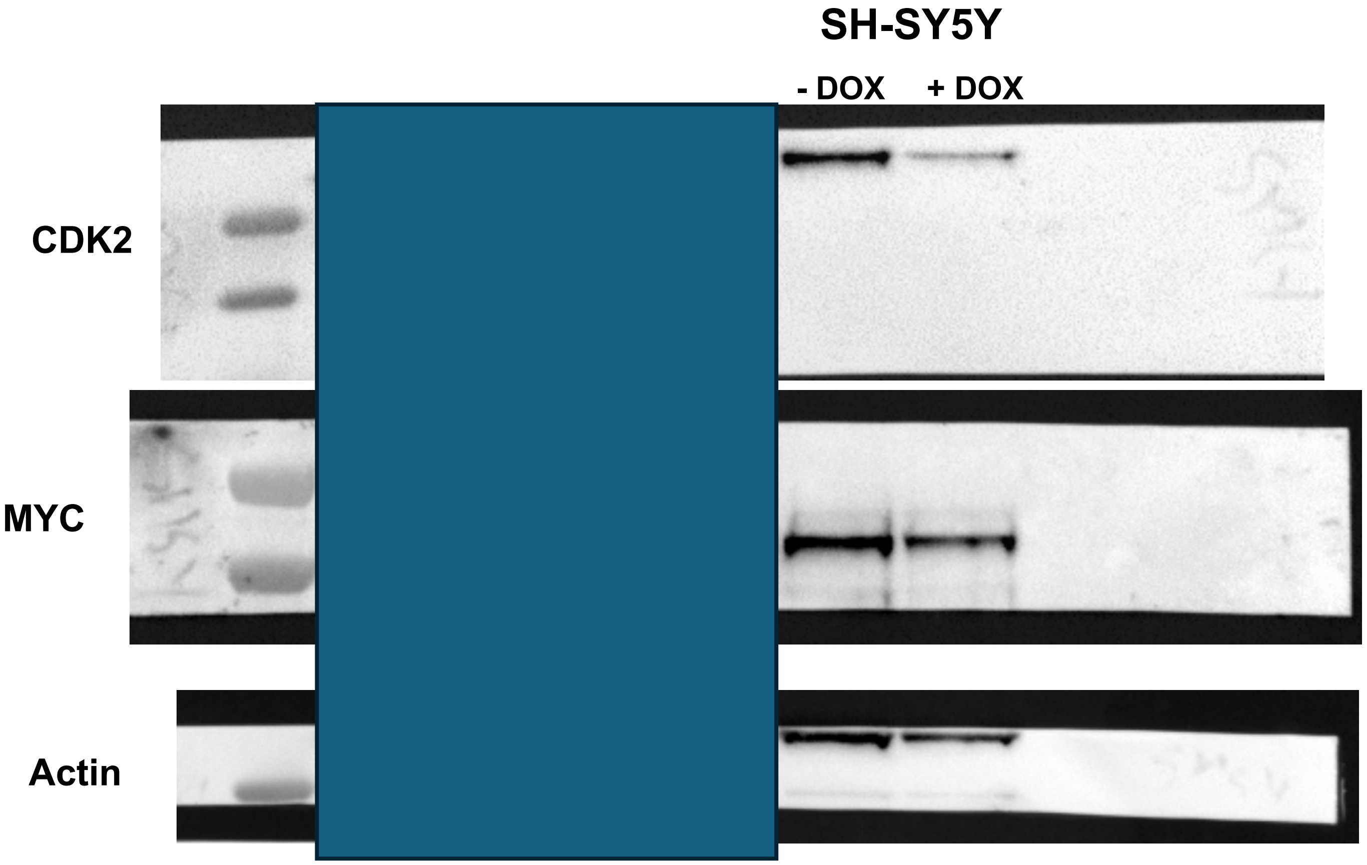

Uncropped western blots related to Figure S6A

SK-N-DZ

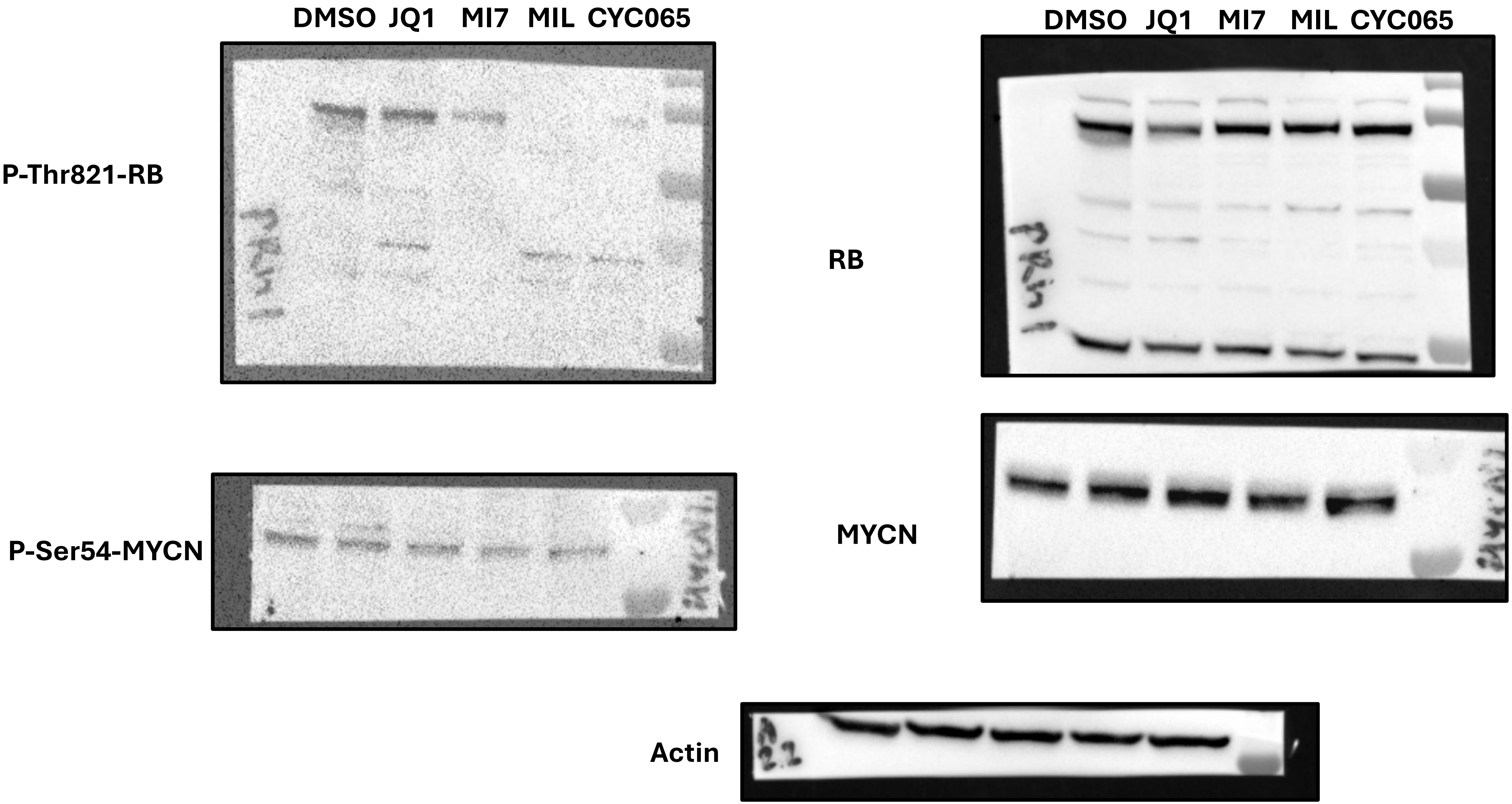

Uncropped western blots related to Figure S6A

SK-N-DZ

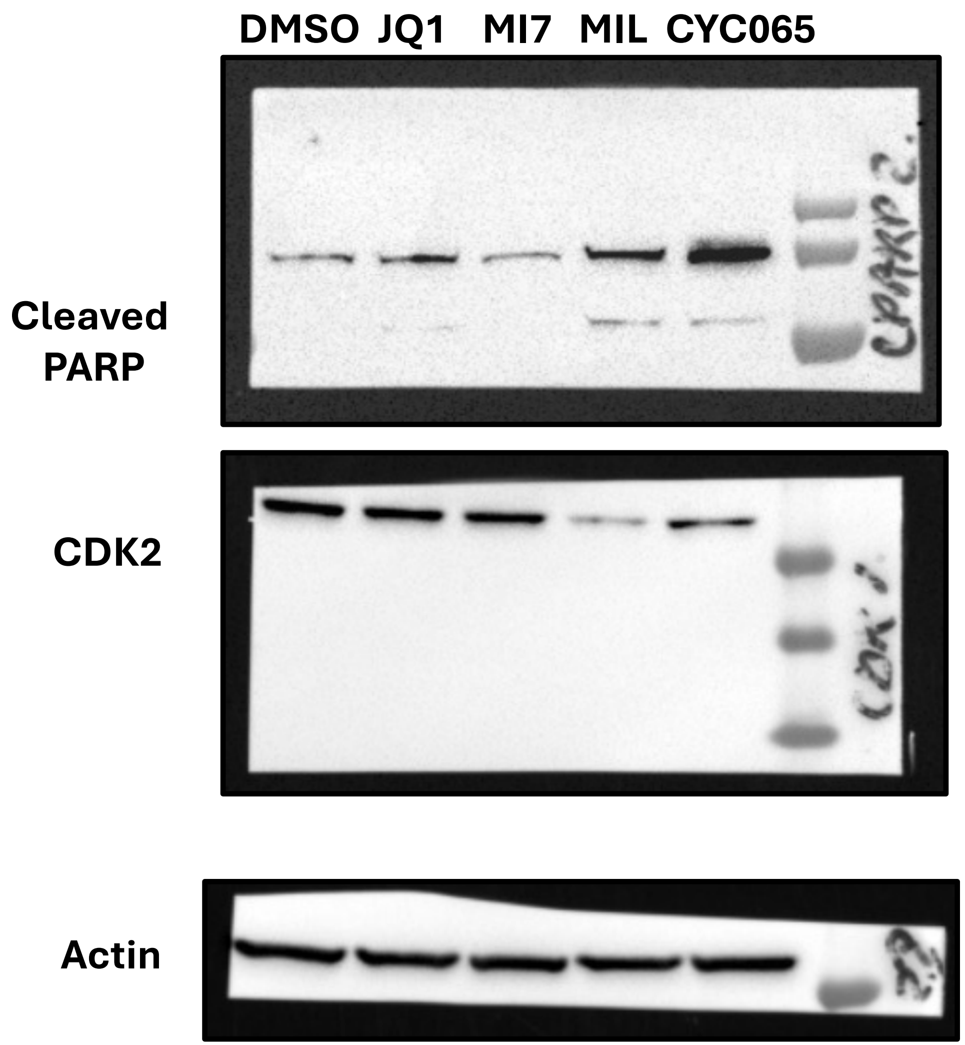

Uncropped western blots related to Figure S6H

IMR32

P-Thr821-RB

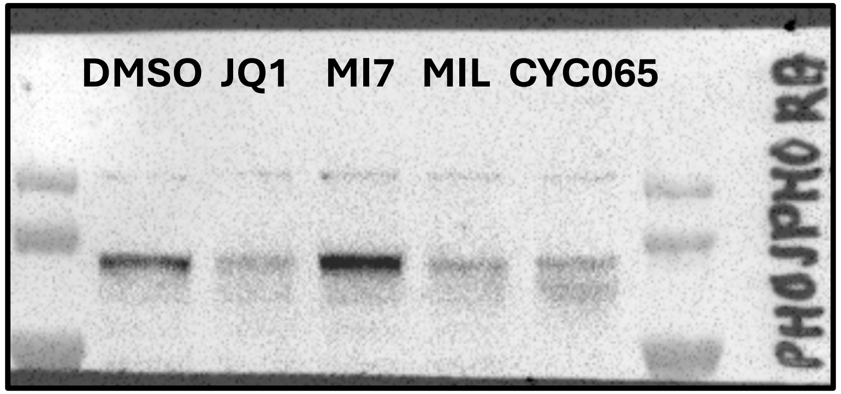

RB

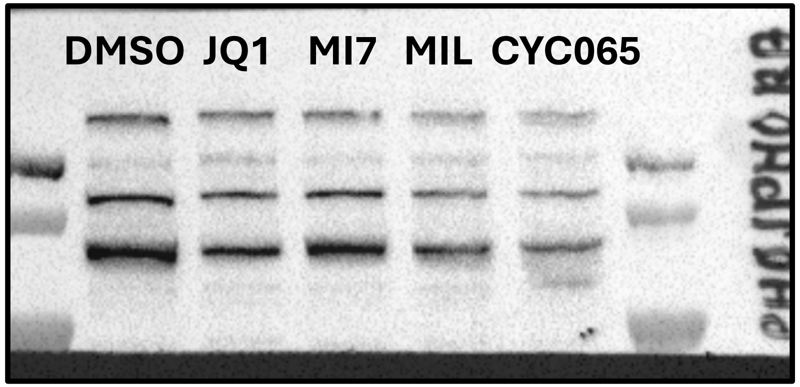

P-Ser54-MYCN

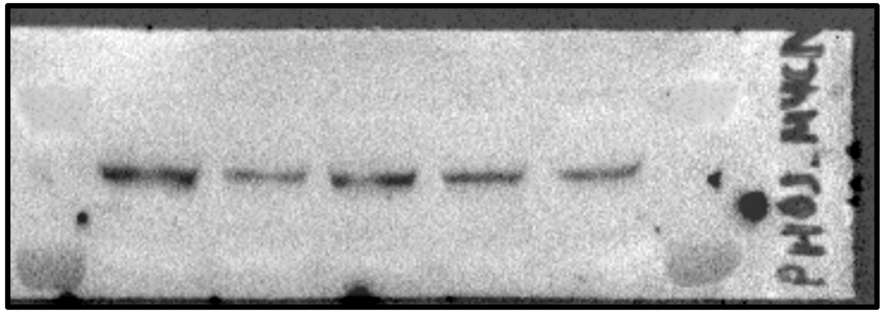

MYCN

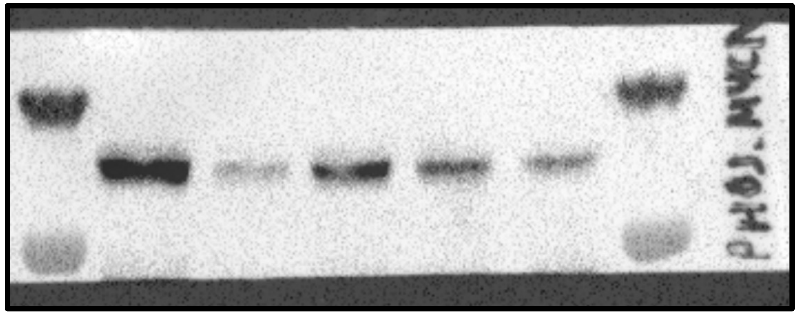

Actin

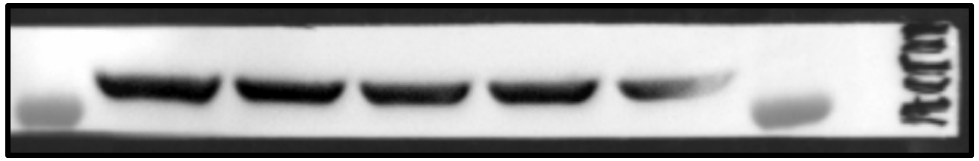

Uncropped western blots related to Figure S6H

IMR32

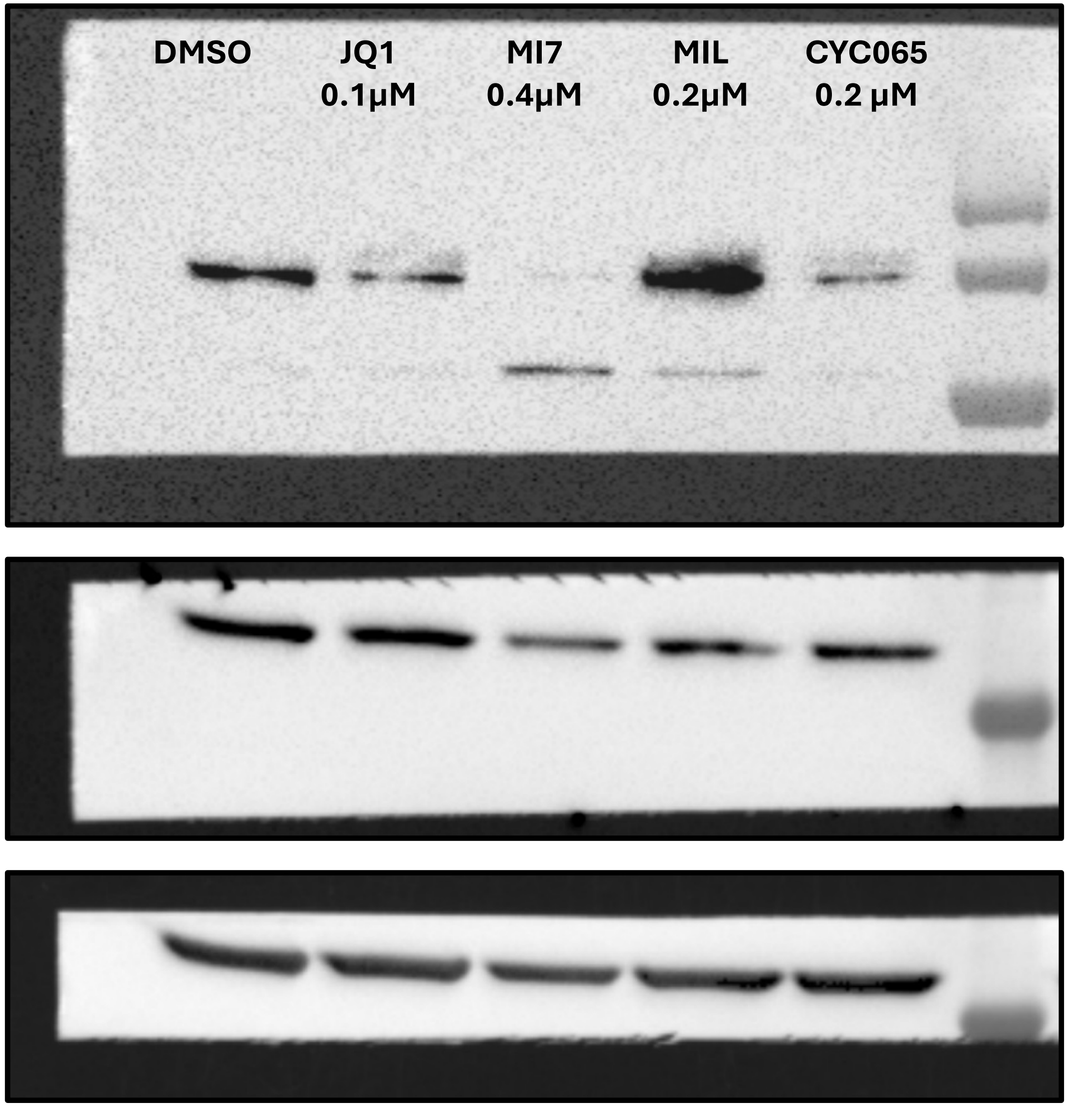

Uncropped western blots related to Figure S7I

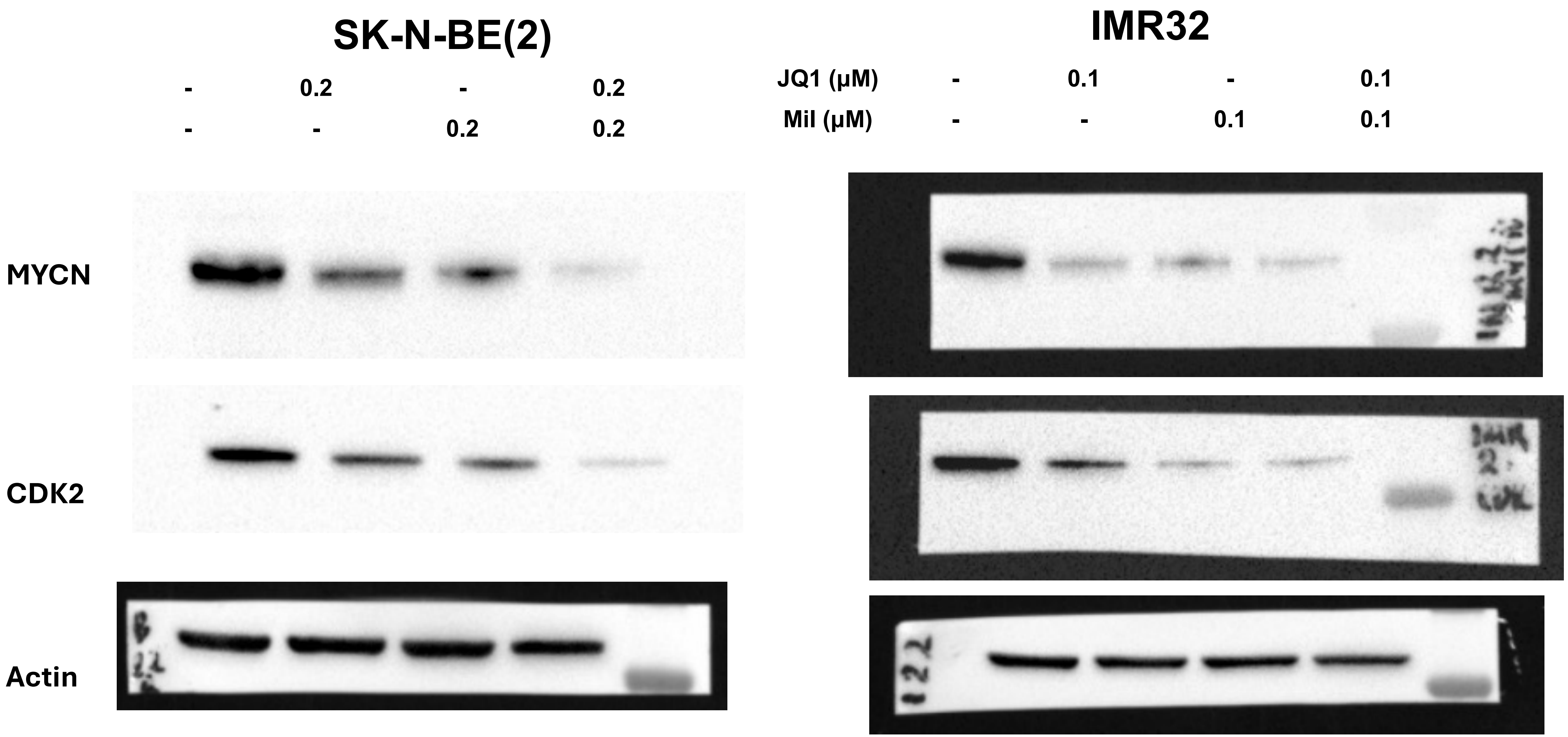

Uncropped western blots related to Figure S8I

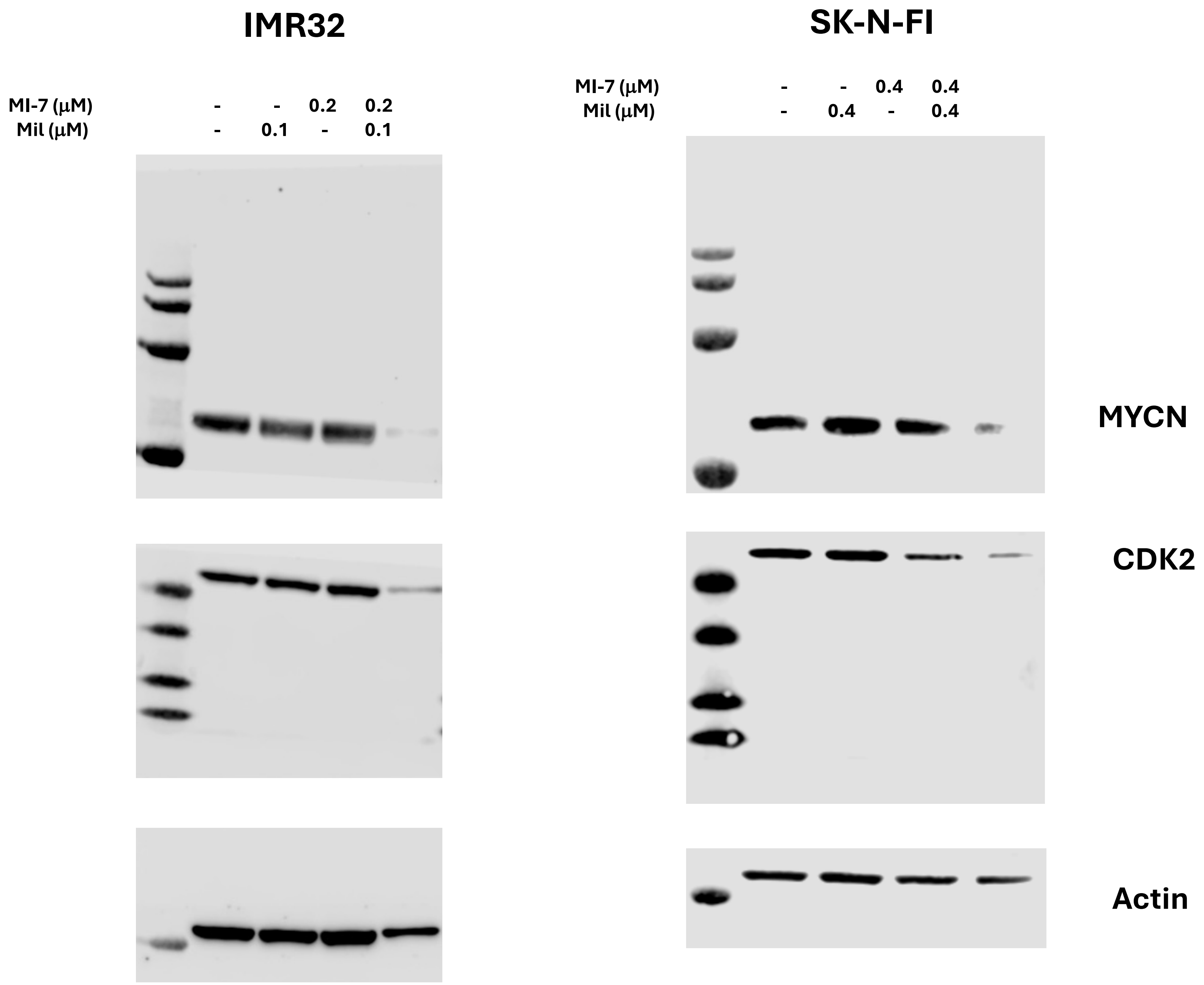

Supplement: Supplementary file 1 — Supplementary Material 1 [file 41598_2026_38123_MOESM1_ESM.pdf]
