# Supplementary material for: CDK2 inhibition promotes neuronal differentiation in neuroblastoma
Source: Sci Rep. 2026 Feb 6;16:5255. doi: 10.1038/s41598-026-38123-4 (PMC12881557; doi:10.1038/s41598-026-38123-4)
Supplement: Supplementary file 3 — Supplementary Material 3 [file 41598_2026_38123_MOESM3_ESM.pdf]

Supplementary Table S2: sequences of qPCR primers used in this study.

Primer	Sequence 5'-3'
GAP43-For	GAGCAGCCAAGCTGAAGAGAAC
GAP43-Rev	GCCATTTCTTAGAGTTCAGGCATG
MEGF8-For	CGGAAAATGGCTTCAACCAGCAG
MEGF8-Rev	CTCGTGGTAGACAGCAGAGTGA
SLIT2-For	CAGAGCTTCAGCAACATGACCC
SLIT2-Rev	GAAAGCACCTTCAGGCACAACAG
STMN2-For	CCAGAAGAAACTGGAGGCTGCA
STMN2-Rev	GCTTTTCCTCCGCCATCTTGCT
SHANK3-For	AGGATCACACCCGCCGAGATTA
SHANK3-Rev	CTACAGACTTGGTCCGTGGAATC
CDK2-For	ATGGATGCCTCTGCTCTCACTG
CDK2-Rev	CCCGATGAGAATGGCAGAAAGC
MYCN-For	ACCACAAGGCCCTCAGTACCTC
MYCN-Rev	TGACAGCCTTGGTGTTGGAGGA
GAPDH-For	TCGGAGTCAACGGATTTGGT
GAPDH-Rev	TGAAGGGGTCATTGATGGCA
